# *trans*-Cyclooctene-caged-IL-1β immunocytokine-constructs ligated to unmodified nanobodies allow click-2-release-based control of cytokine activity†

**DOI:** 10.1039/d5cb00113g

**Published:** 2025-06-04

**Authors:** Amber Barendrecht, Heleen H. C. Peeters, Diana Torres-García, M. Thierry Shema, Alexi J. C. Sarris, Shimrit David, Göktuğ Aba, Camille M. Le Gall, Martin Wilkovitsch, Martijn Verdoes, Hannes Mikula, Mark A. Travis, Sander I. van Kasteren

**Affiliations:** a Division of Chemical Biology and Immunology, Leiden Institute of Chemistry, Universiteit Leiden Gorlaeus Laboratory, Einsteinweg 55 Leiden The Netherlands s.i.van.kasteren@chem.leidenuniv.nl; b Leiden University Medical Center Albinusdreef 2 Leiden The Netherlands; c Department of Medical BioSciences, Radboud University Medical Center Nijmegen The Netherlands; d Institute of Applied Synthetic Chemistry, TU Wien Getreidemarkt 9/163 Vienna Austria; e Institute for Chemical Immunology Nijmegen The Netherlands; f Current address: Department of Immunology, Leiden University Medical Center Leiden The Netherlands; g Manchester Collaborative Centre for Inflammation Research, University of Manchester Manchester UK

## Abstract

Immunocytokines have emerged as a promising modality in cancer therapy, capitalizing on the precision of antibodies to deliver cytokines selectively to tumours. Yet, the toxicity of the cytokine portion of these antibody-cytokine constructs remains a major dose-limiting issue. We present a new approach to control cytokine function without affecting binding of the targeting moiety. By modifying the cytokine with *trans*-cyclooctene carbamates at the lysine positions, we can reduce the binding to the receptor of various highly pro-inflammatory cytokines. Then, using a click-2-release (C2R)-approach, we can reactivate the cytokine activity by reacting it with a variety of tetrazines, through a Diels–Alder-pyridazine-elimination cascade. Finally, we show that the caged cytokines can be conjugated *via* a sortase motif to an unmodified targeting nanobody resulting in a targetable caged immunocytokine construct.

## Abstract 2

Click-triggered reactivation of caged cytokine constructs at the tumour site *via* a Diels–Alder–pyridazine-elimination cascade restores signalling and immune activation. This approach reduces systemic toxicity of immunocytokine therapies.

## Graphical abstract

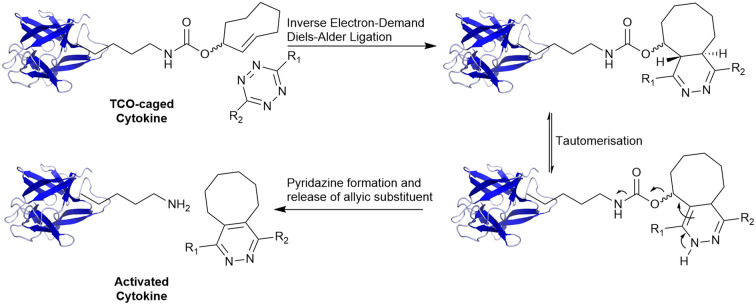

## Introduction

Immunocytokines have emerged as a promising modality in cancer therapy, capitalizing on the precision of antibodies to deliver cytokines selectively to tumours.^1–3^ These protein constructs, first described by Gillies *et al.*, consist of a tumour-targeting antibody moiety linked to an immunological messenger molecule in a single genetic construct.^4^ The initial aim was to open the therapeutic window of the often-toxic cytokine therapies that were in vogue at the time. However, despite their targeted nature, systemic activity remains a major limitation, with on-target toxicity – the cytokine activating the immune system *en route* to a tumour resulting in a cytokine storm – as the key issue.^5,6^ Nevertheless, these targeted therapies have shown promising results in clinical assessments,^7^ albeit with severe side-effects.^8^

Extensive effort has therefore gone into detoxifying immunocytokines to reduce this off-site on-target side-effect profile, including targeted mutagenesis,^9–11^ local activation of the cytokine through dimerization,^12^ and increasing steric hindrance to lower cytokine affinity for its receptor.^13,14^ However, all these current methods are cytokine-specific and require extensive modification of the cytokine, potentially leading to reduced efficacy of the cytokine in the tumour, or even the cytokine being recognised as foreign and being cleared. We envisaged a method whereby the cytokine could be detoxified whilst accumulating in a tumour, and only locally activated, would be a powerful approach to detoxify immunocytokines.

**Fig. 1:**
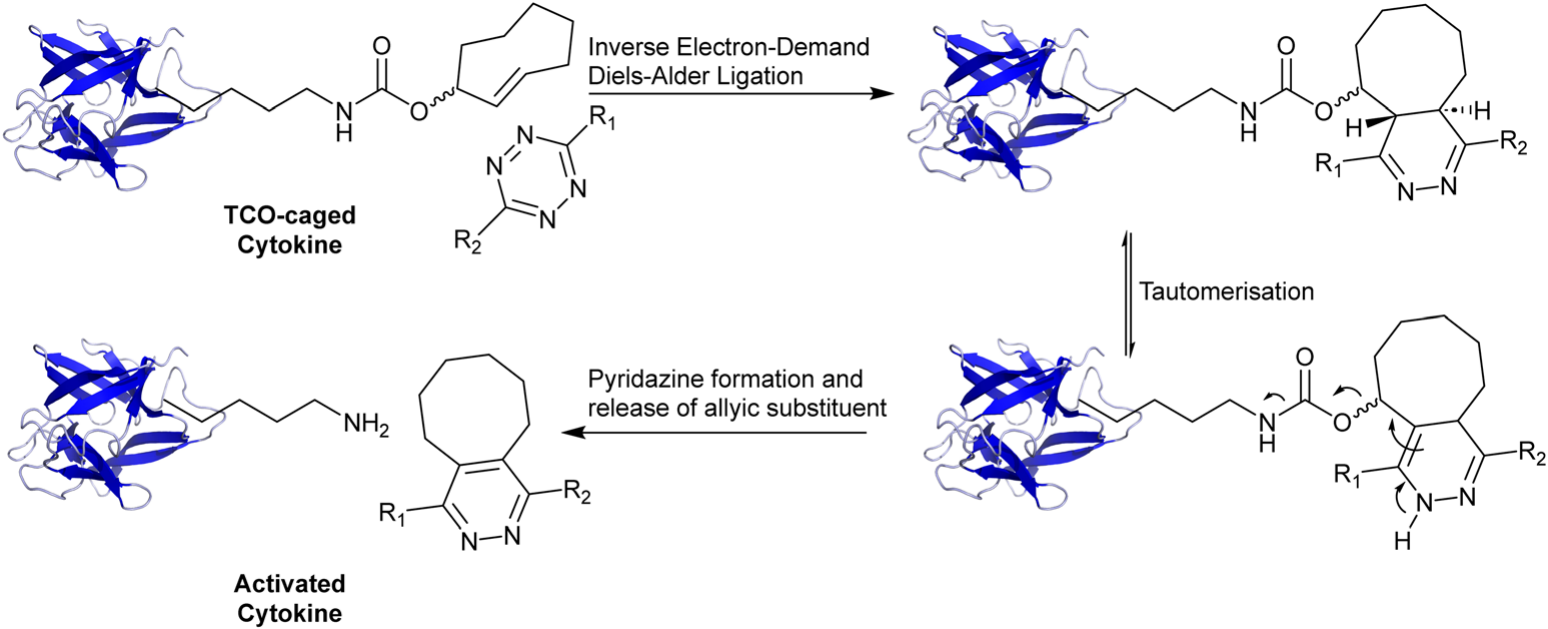
Overview click-2-release approach on a caged cytokine using the inverse electron-demand Diels–Alder reaction. The principle of click-2-release chemistry based on IEDDA with *trans*-cyclooctene (electron-poor) and tetrazine (electron-rich). TCO-caged cytokine reacts with a tetrazine and upon the IEDDA reaction (‘click’) the formed product can release CO_2_ and regains its free amine.

We hypothesized that a click-2-release (C2R) strategy would be a great approach here.^15^ This method, uses the modification of a key heteroatom with a *trans*-cyclooctene group, first reported by Robillard and co-workers^16^ and then by Chen and co-workers,^17^ followed by inducing release with a tetrazine reagent (Fig. 1).^18^ This method has been used extensively to locally activate, and/or deliver prodrugs^19^ through the chemical protection, or ‘caging’ of key heteroatoms, such as amines and alcohols,^20^ followed by controlled deprotection using an inverse electron-demand Diels–Alder (IEDDA) reaction with a tetrazine.^21^ The reason is that the reaction is highly *in vivo* compatible, having been shown in mice,^22^ and now even pursued in human clinical trials.^23^ This method is also employed for the selective activation of chemotherapeutic agents, such as doxorubicin,^16^ and monomethyl auristatin^19^ at tumour sites. The IEDDA reaction enables a rapid and bioorthogonal cycloaddition between *trans*-cyclooctene (TCO) and tetrazine moieties, ensuring that therapeutic agents remain inactive until a tetrazine-based trigger is administered.^24,25^ It is currently even being pursued for the subcellular release of prodrugs^26^ and antigens.^27,28^ We envisaged that this approach would be highly beneficial for detoxifying immunocytokines as the introduction of one,^29^ or in the case of cytokines, multiple TCOs could lead to the blocking of the interaction of the cytokines with their receptors until the point in time of tetrazine addition. We postulated that an amber codon suppression approach^29^ in which a single key lysine is modified would not be of use due to the large contact site between the cytokine and its receptor, as multiple lysine residues are involved in cytokine activity in most cases.

**Fig. 2:**
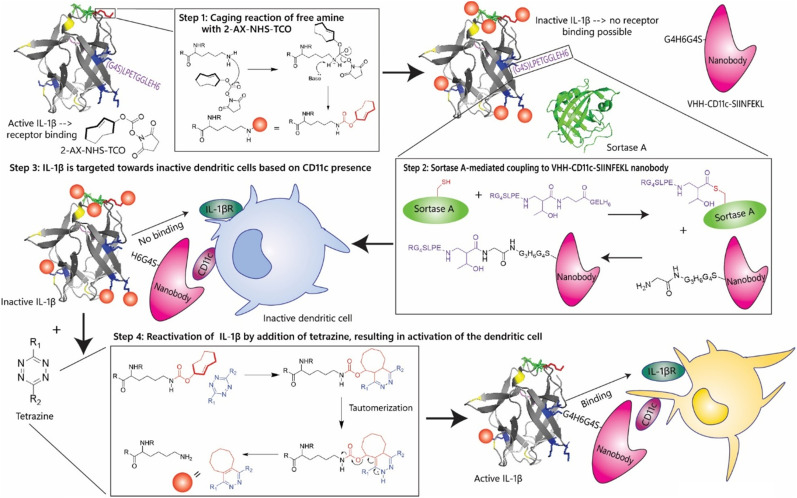
Design of an IL-1β-based immunocytokine using chemical inactivation and reactivation. The first step in the generation of chemically controlled inactivation of immunocytokines. The cytokine (IL-1β) has 15 lysine residues that can be caged using NHS-TCO. Upon caging, the cytokine becomes inactive due to its inability to bind its receptor. The second step is sortase A-mediated coupling the IL-1β cytokine *via* its LPETGG-tag to a nanobody, VHH-CD11c-SIINFEKL with a N-terminal tetra-glycine tail. This allows for targeting of inactive dendritic cells. Inactive IL-1β, unable to interact with its receptor present on the dendritic cell, is treated with a tetrazine, which can remove the TCO-group or ‘cage’ and thereby activate the IL-1β moiety of the immunocytokine. This allows the IL-1β to interact with its receptor (IL-1βR) to activate the dendritic cell in the micro-environment of a tumour to clean up the tumour cells.

Here we describe our first efforts in making caged immunocytokines that can be deprotected after antigen target binding, and their *in vitro* evaluation (Fig. 2). Unlike the only reported caged cytokine where the cytokine is not targeted, but has to be deprotected with an antibody tetrazine conjugate,^30^ we here describe a method in which we first cage the cytokine portion of an immunocytokine and conjugate it to an uncaged targeting moiety (a nanobody in this case) using a sortase-based approach. Such an approach yielded a caged immunocytokine in which the targeting was not affected by the caging, and only the function of the cytokine could be controlled. We also found that by lowering the temperature of the caging reaction, IL-1β, IL-2, TNF-α, and IFN-γ could be caged in a manner that allowed recovery of function after uncaging. Interleukin 2 (IL-2) and TNF-α are the most extensively investigated cytokines for immunocytokine production, due to their high toxicity and high potential clinical benefit in treating tumours.^31^ Immunocytokines based on these proteins have been used in the treatment of *e.g.* metastatic melanoma.^32,33^ Interferon gamma (IFN-γ), is critical for antitumour responses, and directly suppresses tumourigenesis, shown by heightened tumour susceptibility in IFN-γ-deficient models.^34,35^ IL-1β-based immunocytokines are a particular interest, as they not only have potentially toxic on-target off-tumour effects, but also have been reported to play controversial roles in cancer, abetting both development^36–41^ and tumour regression.^42,43^

IL-1β is highly amenable to a caging strategy as it has 6 surface-exposed lysine residues (K88, K92, K93,K94) that are involved in its receptor binding (Fig. S1, ESI†).^44,45^ IL-1β is produced as an inactive 31 kDa pro-protein and undergoes proteolytic cleavage to generate the active 17 kDa form.^46^ IL-1β interacts with two receptors: IL-1 receptor I (IL1RI), which triggers a pro-inflammatory signalling cascade *via* NF-κB activation, and IL-1 receptor II (IL1RII), which serves as a decoy receptor and inhibits downstream signalling.^43,47,48^ IL-1β is known to be involved in the progression of multiple myeloma^49^ as it causes the production of IL-6 which causes the development of diseased plasma cells.^50^ Contradictory to these findings, it has been found that mice with SA1 sarcoma benefit from injections with external IL-1β, causing the regression of the tumour.^51^ Its capacity to drive inflammation and its association with secondary cytokine release have made its systemic administration particularly challenging.^45^ The use of IL-1β in immunocytokine formats has been explored, including monoclonal antibodies F8 (specific to the alternatively-spliced extra-domain A domain of fibronectin, a marker of tumor angiogenesis) designed to selectively deliver IL-1β to tumour stromal components.^52^ However, despite the potential for improved targeting, these constructs have exhibited unacceptable systemic toxicity, ultimately preventing their clinical development. We therefore started by determining whether the activity of IL-1β could be controlled through caging with a TCO-modality in such a manner that it could be reactivated upon treatment with tetrazine.

## Results and discussion

The first step in obtaining an immunocytokine with a caged cytokine moiety was to identify a caging strategy that effectively reduces immunocytokine activity upon NHS-TCO-caging,^53^ in such a way that activity could be restored upon click-2-release reaction. This means – particularly as the C2R-reaction is not 100% efficient – that the caging/decaging conditions required extensive optimisation to prevent structural changes or protein misfolding due to the caging, whilst at the same time ensuring the blocking of enough of the interaction of the cytokine with its receptor(s) that function was obstructed. And all this in such a manner that activity could be restored upon reaction with tetrazine.

This initial optimisation was performed using murine IL-1β as a model cytokine due to its well-characterized structure and high lysine content near the receptor-binding interface.^54^ This made this cytokine particularly suitable for lysine-targeted modification strategies. Initial caging experiments were performed with different concentrations of NHS-TCO in 20 mM HEPES buffer (pH 8) and caging efficiency was assessed by an IL-1β-specific ELISA (Fig. S2A–C, ESI†).^54^ Increasing NHS-TCO concentrations indeed reduced ELISA signal (Fig. S2A, ESI†), however, incubation beyond 1 hour analysis resulted in precipitation of the protein (as measured by SDS-PAGE, Fig. S2B, ESI†). Optimal conditions for caging (not taking decaging into account) were found to be incubation 1 hour with between 4.0–8.0 mM NHS-TCO at 37 °C (Fig. S2C, ESI†).

**Fig. 3:**
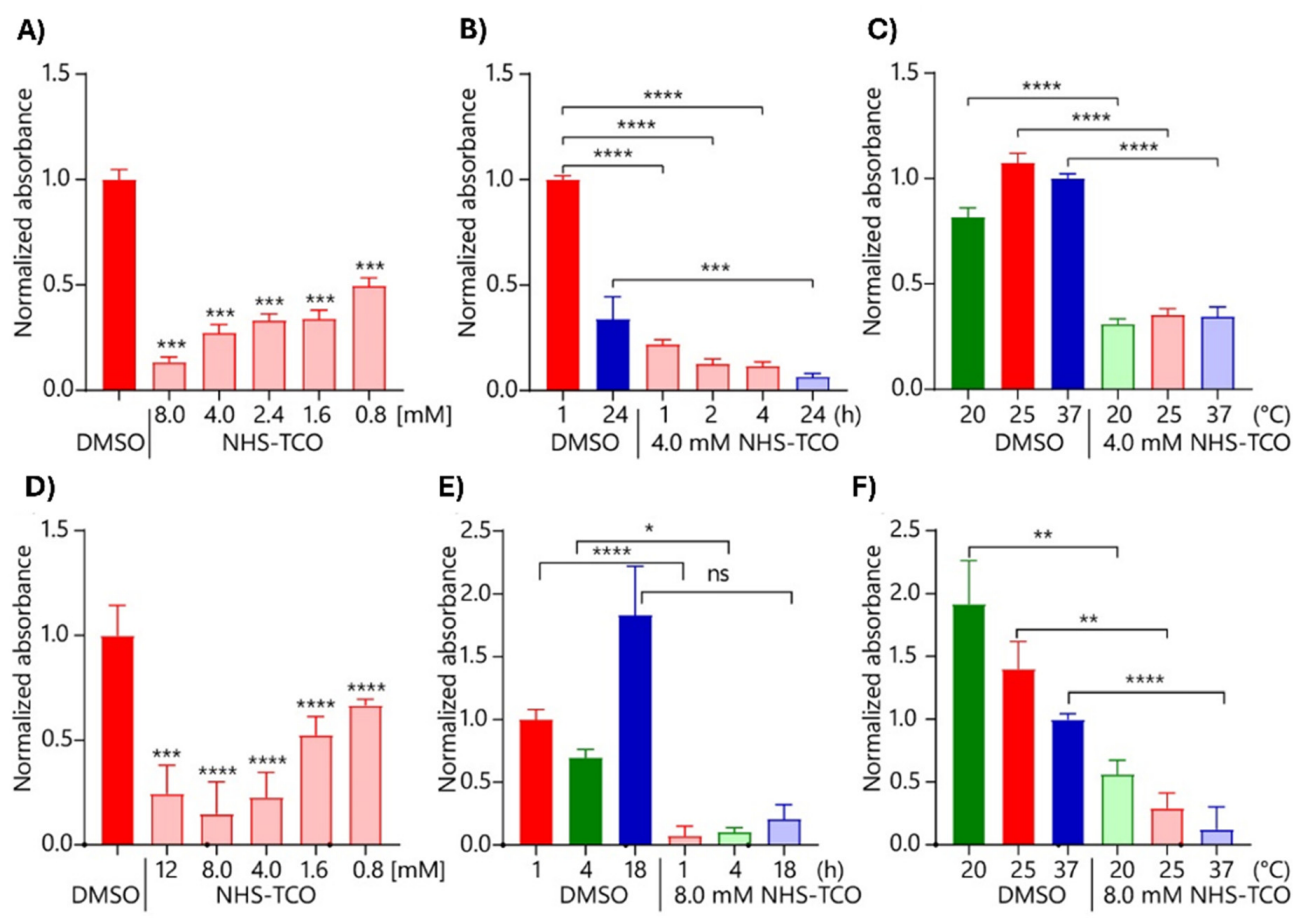
Analysis of caging optimisation of IL-1β on HEK-Blue IL-1β cells and RAW-Blue cells using QUANTI-Blue colorimetric assay. (A)–(C) The caging reaction using NHS-TCO was assessed using (caged) cytokine stimulated HEK-Blue IL-1β. (A) Analysis of caging with increasing concentration of NHS-TCO (*N* = 4, *n* = 8); (B) analysis of caging time (h) (*N* = 4, *n* = 8); (C) optimisation of the caging temperature (°C) (*N* = 3, *n* = 6). (D)–(F) The caging reaction using NHS-TCO was assessed using (caged) cytokine stimulated RAW-Blue. (D) Analysis of caging with increasing concentration of NHS-TCO (*N* = 3, *n* = 6); (E) analysis of caging time (h) (*N* = 3, *n* = 6); (F) optimisation of the caging temperature (°C) (*N* = 3, *n* = 6). Bright colours indicate DMSO only samples and pastels the TCO-treated samples. Colour groups indicate different conditions with respective DMSO controls. Data were plotted as mean signal ± SEM. Significances are indicated as follows: *P < 0.05; **P < 0.01; ***P < 0.001; ****P < 0.0001; ns is non-significant.

To determine whether TCO-modification also affected IL-1β-receptor binding, HEK-Blue IL-1β and RAW-Blue assays were performed.^55,56^ HEK-Blue cells detect cytokine activity *via* NF-κB-mediated SEAP expression, quantified using QUANTI-Blue.^56–58^ RAW-Blue cells are murine macrophages that are also engineered to express SEAP upon NF-κB activation.^55,59^ Colorimetric evaluation of SEAP activity therefore allowed the quantitative assessment of IL-1β-receptor activation by the TCO-modified IL-1β construct.^58^ Both the HEK-Blue IL-1β-assay (Fig. 3A–C) and the RAW-Blue assay (Fig. 3D–F) showed that increasing the NHS-TCO concentration during the modification reaction, resulted in a product that was less capable of activating these cells (Fig. 3A and D). The highest concentration of NHS-TCO (8 mM) reduced the activity to 10% of the original value. Under 18-hour control conditions, IL-1β activity increased non-significantly, but still unexpectedly (Fig. 3E), whereas extended incubation (24 hours; Fig. 3B) results in decreased activity. These observations suggest that prolonged single-protein incubation at 37 °C may induce protein folding changes that alter cytokine activity, potentially contributing to variability in experimental outcomes. Lowering the caging temperature did not affect caging efficiency (Fig. 3C and F).

To correlate IL-1β activity loss to lysine modification levels, ESI LC-MS was performed on caged samples (Fig. S3A–G, ESI†). For sufficient signal, protein concentration was increased to 11 μM. Murine IL-1β contains 15 surface-exposed lysines, of which 14 are accessible for modification.^54^ LC-MS analysis showed that caging with 8.0 mM NHS-TCO resulted in an average of 7–8 modified lysines (Fig. S3B, ESI†), while 1.6 mM NHS-TCO reduced this to 4 (Fig. S3A, ESI†). Increasing NHS-TCO to 16 mM did not raise the average modification level but decreased the proportion of minimally modified species (Fig. S3B and C, ESI†).

**Fig. 4:**
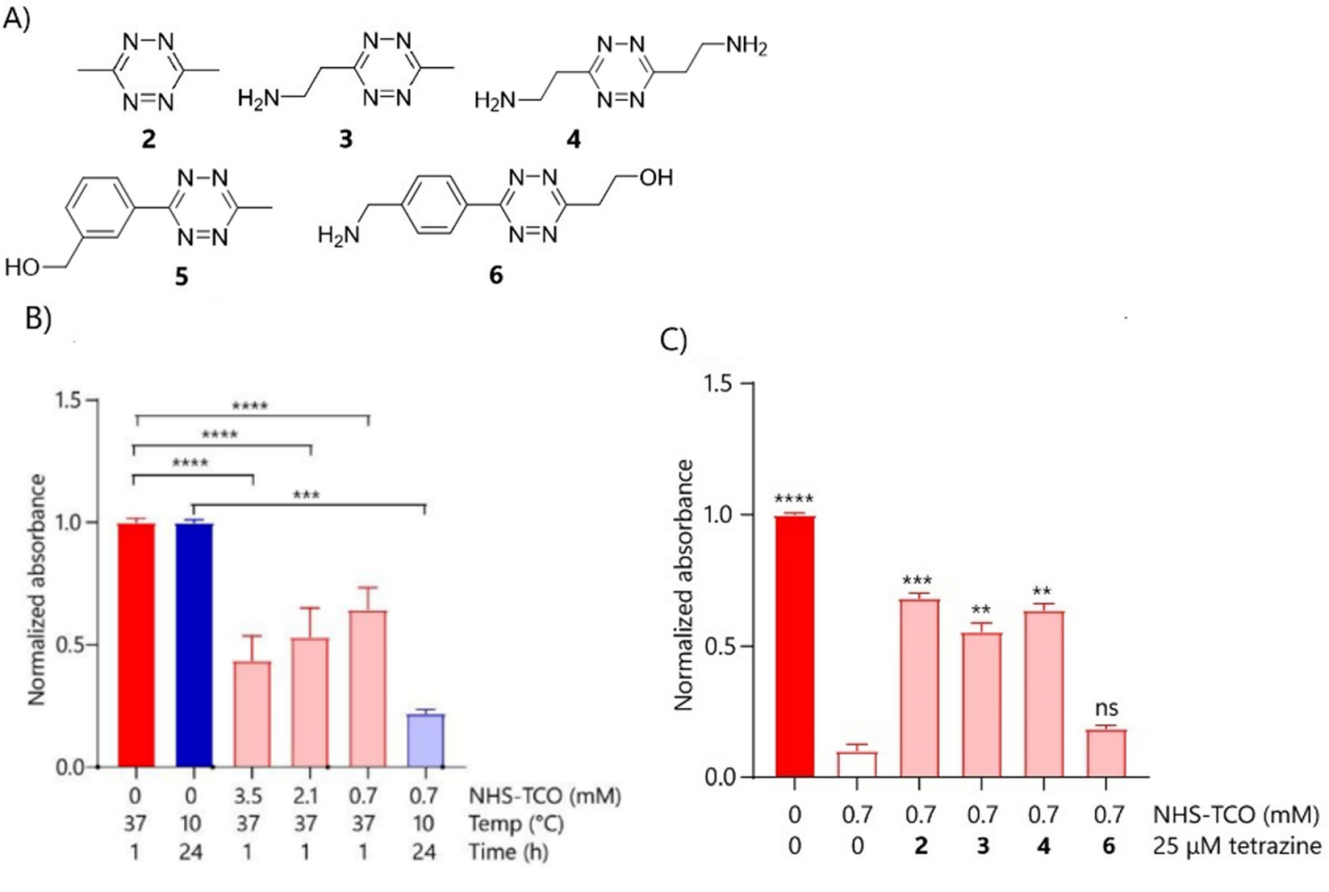
Initial decaging optimisation of IL-1β and IL-1β-LPETGG using different tetrazines analysed by RAW-Blue cells and HEK-Blue IL-1β cells, respectively. (A) Various tetrazines tested for decaging of IL-1β-LPETGG. (B) IL-1β-LPETGG (5 μM) was caged in 20 mM HEPES with either 3.5 mM, 2.1 mM or 0.7 mM NHS-TCO for 1 hour at 37 °C or with 0.7 mM NHS-TCO for 24 hours at 10 °C. The caged protein was assessed by HEK-Blue IL-1β (*N* = 5, *n* = 10). (C) IL-1β-LPETGG (5 μM) was caged with 0.7 mM NHS-TCO for 24 hours at 10 °C in 20 mM HEPES pH 8, following addition to HEK-Blue IL-1β cells in the applicable concentration. Decaging was performed with 25 μM of different tetrazines and took place for 18–20 hours at 37 °C on the cells (*N* = 6, *n* = 12). Bright colours indicate DMSO only samples and pastels the TCO-treated samples. Colour groups indicate different conditions with respective DMSO control. Data were plotted as mean signal ± SEM. Significances are indicated as follows: *P < 0.05; **P < 0.01; ***P < 0.001; ****P < 0.0001; ns is non-significant.

It was next determined whether any of the above-caged proteins could also be reactivated upon reaction with tetrazines. And here the project hit a snag: initial decaging experiments (Fig. S4A–C, ESI†) showed that IL-1β could not be restored to its functional form for any of the above caging levels. This may be due to incomplete removal of TCO groups or irreversible inactivation due to protein unfolding during caging. We postulated that, given that IL-1β has 14 accessible lysines^44,60^ extensive modification at elevated temperatures and high NHS-TCO concentrations could lead to the disruption of the protein fold, leading to an irreversible loss of activity. In an attempt to address this, we assessed whether modifications at lower temperatures could prevent this permanent deactivation during the TCO reaction. IL-1β was therefore modified with 3.5 mM, 2.1 mM or with 0.7 mM NHS-TCO at 10 °C for 24 h (Fig. 4B). HEK-Blue IL-1β confirmed that caging with this lower NHS-TCO concentration at the lower temperature/longer reaction time resulted in similar reduction of IL-1β (Fig. 4B). Using these caging conditions, decaging was tested in the HEK-Blue IL-1β assay using tetrazines (2–6, Fig. 4A and C). Tetrazines 2–4 yielded significant recovery of IL-1β activity in this assay, with 3,6-dimethyl-tetrazine 2 showing the highest IL-β activity recovery of approximately 60%.

**Fig. 5:**
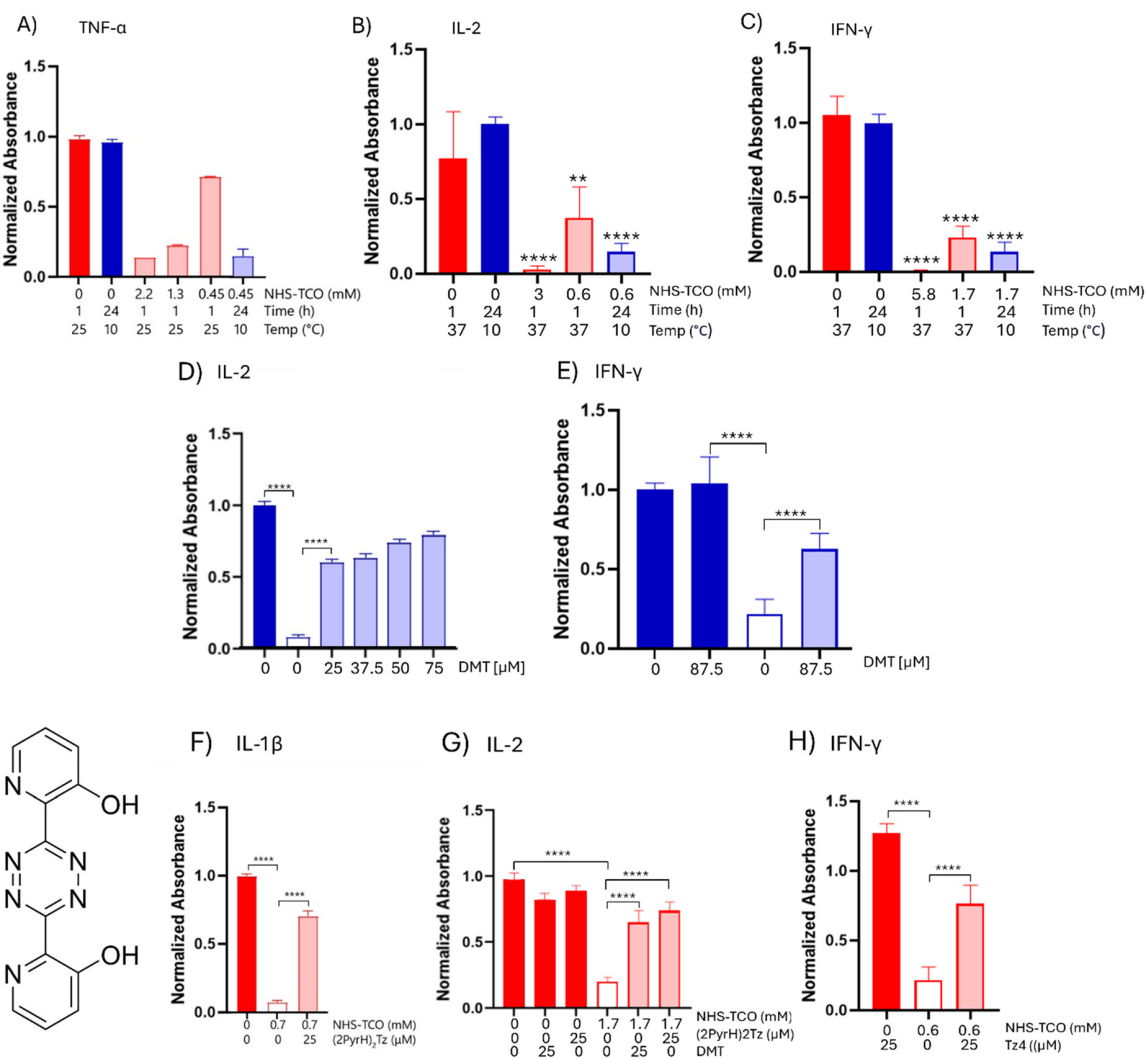
Caging at lower temperatures of TNF-α-LPETGG-6His, IL-2-LPETGG-6His and IFN-γ and decaging optimisation. (A) TNF-α-LPETGG-6His (0.1 mg mL^−1^ or 5.1 μM) in 20 mM HEPES pH 8 was caged either at 25 °C for 1 hour with 0.47–2.2 mM NHS-TCO or at 10 °C for 24 hours with 0.05–0.45 mM NHS-TCO. The caging was analysed with HEK-Blue TNF-α cells (*N* = 1, *n* = 2). (B) IL-2-LEPTGG-6His (0.1 mg mL^−1^ or 5.6 μM) in 20 mM HEPES pH 8 was caged either at 37 °C for 1 hour with 0.6 or 3 mM NHS-TCO or at 10 °C for 24 hours with 0.3 mM NHS-TCO. The caging was analysed with HEK-Blue IL-2 cells (*N* = 3, *n* = 6). (C) IFN-γ (0.1 mg mL^−1^ or 5.9 μM) in PBS was caged either at 37 °C for 1 hour with 1.7 or 5.8 mM NHS-TCO or at 10 °C for 24 hours with 1.7 mM NHS-TCO. The caging was analysed with THP-1-DUAL cells (*N* = 4, *n* = 8). (D) The reactivation of IL-2 caged with 0.6 mM NHS-TCO (for 24 hours at 10 °C) using different concentrations of DMT (*N* = 4, *n* = 8) analysed in HEK-Blue IL-2 cells. (E) The reactivation of IFN-γ caged with 1.7 mM NHS-TCO (for 24 hours at 10 °C) using 87.5 μM DMT (*N* = 4, *n* = 8) analysed in THP-1-DUAL cells. (F) The reactivation of IL-1β-LPETGG, caged with 0.7 mM NHS-TCO (for 24 hours at 10 °C) (*N* = 5, *n* = 10) analysed in HEK-Blue IL-1β cells. (G) The reactivation of IL-2 caged with 0.6 mM NHS-TCO (for 24 hours at 10 °C) (*N* = 4, *n* = 8) analysed in HEK-Blue IL-2 cells. (H) The reactivation of IFN-γ caged with 1.7 mM NHS-TCO (for 24 hours at 10 °C) (*N* = 4, *n* = 8) analysed in THP-1-DUAL cells. Caging was compared with the DMSO control which gave the maximum signal. Bright colours indicate DMSO only samples and pastels the TCO-treated samples. Colour groups indicate different conditions with respective DMSO controls. Data were plotted as mean signal ± SEM. Due to the limited number of datapoints no significances could be determined in A, for other graphs significances are indicated as follows: *P < 0.05; **P < 0.01; ***P < 0.001; ****P < 0.0001; ns is non-significant.

Following promising caging and decaging results with IL-1β, the strategy was extended to a sortaggable IL-1β variant expressed from a pET28a(+) vector, which showed comparable behaviour to wild-type IL-1β. The approach was also applied to therapeutically relevant cytokines, TNF-α, IL-2, and IFN-γ. IL-2 (K35, K43 and K64)^61^ and IFN-γ (K108, K125, K128 and K130)^62^ contain lysine residues critical for receptor binding. For TNF-α this is more complex, as no lysine residues are directly involved in receptor binding or trimerization. However, certain lysine residues are critical for proper TNF-α (K112) folding, and their modification may impair the structural integrity of the cytokine, thereby indirectly reducing its affinity for TNF receptors (TNFRs).^63^ Initial caging experiments at 37 °C again resulted in proteins that could not be efficiently reactivated by tetrazine treatment (Fig. S5A–I, ESI†). However, like for IL-1β, the cold caging strategy with low-temperature incubations with reduced NHS-TCO concentrations resulted in efficient inactivation for all cytokines (Fig. 5A–C). Decaging with tetrazine 2 of TNF-α, IL-2 and IFN-γ, yielded some promising results: IL-2 function was restored to 90% at a concentration of 75 μM 2 (Fig. 5D). IFN-γ function was restored to 60% at a concentration of 25 μM 2 (Fig. 5E), increasing demethylsufide (DMT, 2) concentration did not result in more sufficient decaging (data not shown). Decaging of TNF-α (Fig. S6A, ESI†) restored only 20% compared to caged cytokine activity. Further optimisation of TNF-α caging did not improve either the caging, nor the decaging activity. A novel tetrazine, 2,2′-(1,2,4,5-tetrazine-3,6-diyl)bis(pyridin-3-ol) ((2PyrH)_2_Tz),^64^ outperformed other tetrazines for IL-1β and IL-2 decaging (Fig. 5F and G), but failed to restore TNF-α activity (Fig. S6B, ESI†). Moreover, higher concentrations of (2PyrH)_2_Tz induced cytotoxicity (data not shown). Decaging of IFN-γ using (2PyrH)_2_Tz was inconclusive due to high background signal in the Quanti-Luc assay. However, a test with Tz4, that showed promising decaging for IL-1β, restored activity of IFN-γ to approximately 75% of the original activity (Fig. 5H). It may be valuable to explore alternative tetrazine designs similar to those described by Fan *et al.* (2016).^65^ In this work, the authors introduced a series of unsymmetrical tetrazines, optimized with an electron-withdrawing group (EWG) at the 3-position to accelerate the initial cycloaddition with TCO, and a small, non-EWG group at the 6-position to facilitate the subsequent elimination (release) step. Their best-performing derivative achieved >90% decaging efficiency in living cells within 4 minutes, highlighting the potential of such structural designs to further enhance bioorthogonal decaging performance.

Building on the successful control of cytokine activity *via* click-2-release chemistry, the next objective was to generate an immunocytokine by conjugating the caged cytokine to a tumour-targeting moiety. While immunocytokines are typically produced *via* genetic fusion, this strategy is incompatible with non-selective lysine modification, as it would indiscriminately modify both the cytokine and targeting domain. Although this would result in the desired loss of cytokine activity, it would also result in undesired loss of antigen binding capacity. The targeting-reagent therefore had to be coupled to the cytokine only after it had been modified with TCO-carbamates. To achieve this, it was decided to use a sortase A-based approach in which a small single-chain fragment of a camelid antibody was genetically modified with an N-terminal tag and the cytokine with a C-terminal tag (LPETG). Sortase A from *Staphylococcus aureus* catalyses the ligation of a LPETG-motif and a poly-glycine tail in peptide–protein^66^ and protein–protein^67,68^ systems. This strategy was employed to ligate a TCO-modified cytokine carrying a *C*-terminal LPETGG-tag to a nanobody equipped with an N-terminal glycine tag. Reported ligation yields (40–85%), would be sufficient to produce caged immunocytokines for further evaluation.

**Fig. 6:**
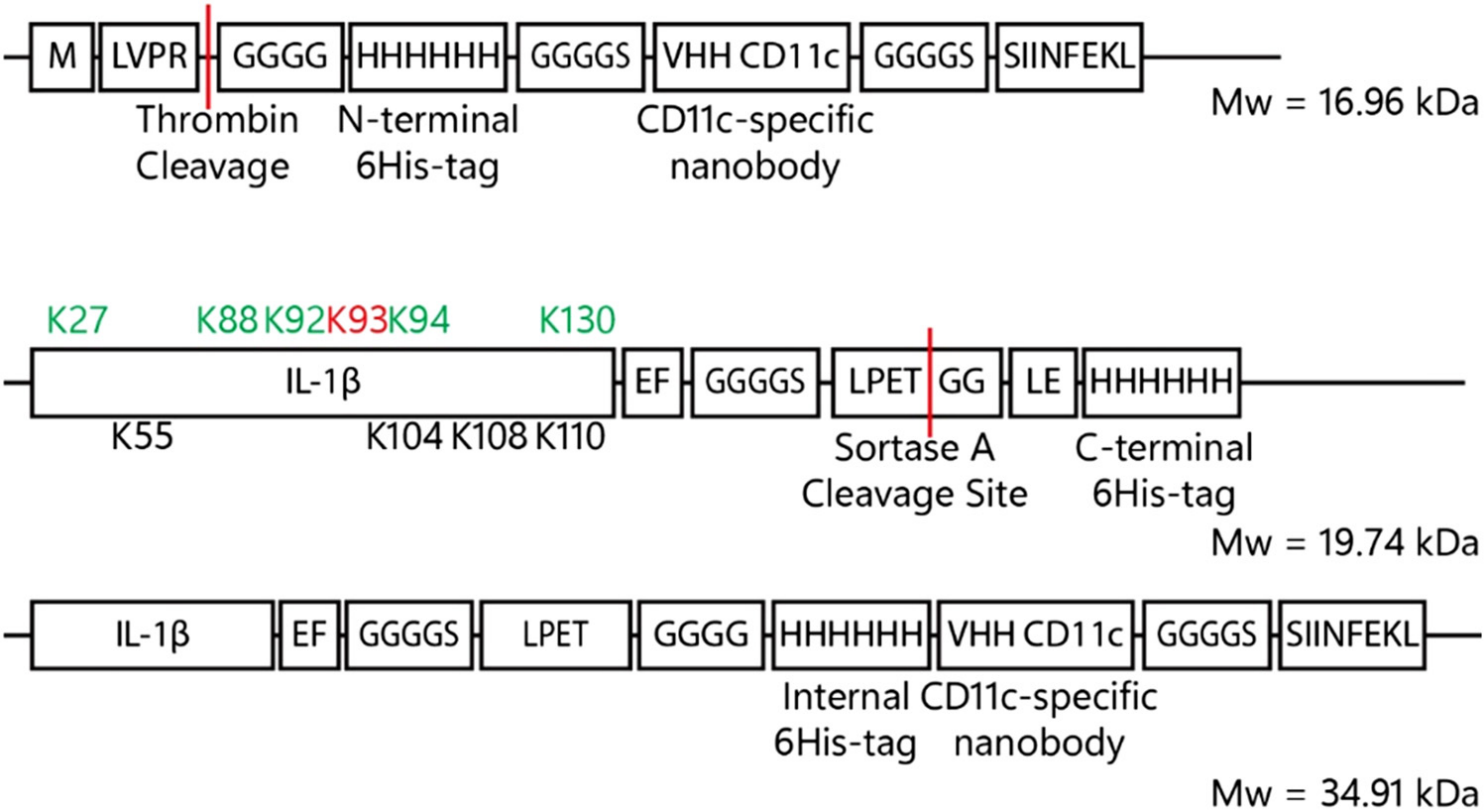
Construct formation VHH-CD11c, IL-1β-LPETGG-6His and the final product after sortase A ligation. The VHH-CD11c nanobody consists – from N- to C-terminus of thrombin-tag-protected tetra glycine N-terminus followed by a 6His-tag for purification purposes which is fused to the actual nanobody *via* a GGGGS-spacer. The C-terminus contains a SIINFEKL peptide for potential T-cell activation. IL-1β is C-terminally fused to LPETGG, the sortase A motif, *via* a GGGGS-spacer. For purification purposes, a C-terminal 6His-tag was introduced which is removed upon coupling to the VHH-CD11c-SIINFEKL nanobody. The green coloured lysines are involved in receptor interaction and the black lysine residues are adjacent to receptor interacting residues. The red coloured lysine has important hydrophilic interactions. Upon reaction with sortase A and the VHH-CD11c nanobody, the C-terminal 6His-tag is replaced by the CD11c-specific nanobody by a transpeptidation reaction.

In order to obtain the immunocytokine in which the cytokine was inactive, a construct consisting of a nanobody targeting group is used, linked by a sortase reaction to the murine IL-1β-gene construct. The choice was made to use a nanobody instead of an antibody as a targeting group. Until now immunocytokines were based on intact antibodies (IgGs)^67,69,70^ or single chain variable fragments (scFv).^71–77^ However nanobodies were selected over full-length antibodies or scFvs due to their smaller size, thermal and pH stability, lack of glycosylation and hydrophobic domains, and efficient bacterial expression.^78,79^ IL-1β was modified with the sortase motif at the *C*-terminus for two reasons: first, N-terminal TCO-modification would preclude sortase ligation; second, N-terminal modification has been reported to reduce IL-1β activity.^80^ The IL-1β gene (Gene ID: 16176) was engineered with a *C*-terminal GGGGS spacer, followed by the LPETGG sortase recognition motif and a 6His-tag for purification (Fig. 6).^81–84^

To obtain IL-1β-LPETGG-6His the sequence was expressed from pET28a(+)-vector using *E. coli ArcticExpress* (DE3) RP system, which can express proteins with rare codons.^85,86^ Expression was induced with 0.5 mM IPTG under T7 promoter control, and carried out at 10 °C for 3 days (Fig. S7A, ESI†).^87^ Lysis was performed with lysozyme and sonication, followed by purification from the soluble fraction *via* nickel affinity chromatography (Fig. S7b, ESI†). Sortase A was expressed from the pET28aSrtAΔ59-expression vector as previously described.^87^ The anti-CD11c nanobody or VHH-CD11c modified with a pelB signal sequence was expressed from pET22b-vector in *E. coli* BL21 (DE3) pLysS as reported.^88^

**Fig. 7:**
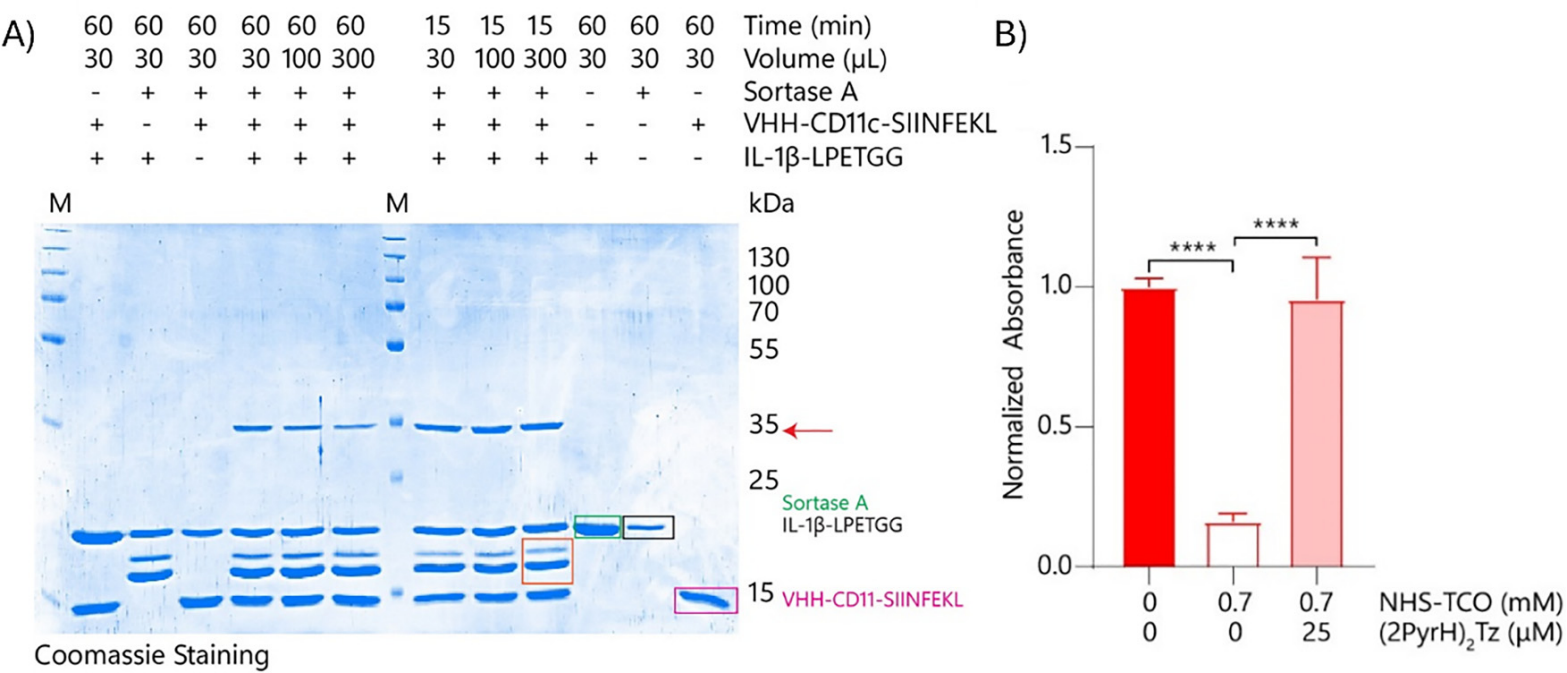
Sortase A reaction optimisation of reaction volume and time and decaging evaluation of IL-1β-VHH-CD11c-SIINFEKL using HEK-Blue IL-1β cells. (A) Sortase A (3 μM, 19 kDa green box) reaction with IL-1β-LPETGG (4 μM, 19.7 kDa black box) and VHH-CD11c-SIINFEKL (8 μM, 15 kDa pink box) was optimised and analysed using SDS-PAGE. The reaction was performed in 50 mM Tris pH 7.5/150 mM NaCl/10% (v/v) glycerol supplemented with 1 mM CaCl_2_. Both reaction time and reaction volume were optimised. Maximal product formation was realised with 15 minutes reaction time in 100–300 μL reaction volume. The red arrow shows the 35 kDa conjugate, the orange box circles the hydrolysed products from IL-1β reacting with Sortase A. (B) The re-activation of IL-1β-VHH-CD11c-SIINFEKL (5.0 μM) by (2PyrH)_2_ Tz was analysed on HEK-Blue IL-1β cells. Caging was performed before coupling to VHH-CD11c-SIINFEKL, in 20 mM HEPES pH 8 for 2.1 mM NHS-TCO (for 1 hour at 37 °C) (*N* = 5) bright colours indicate DMSO only samples and pastels the tetrazine treated samples. Data were plotted as mean signal ± SEM. Significances are indicated as follows: *P < 0.05; **P < 0.01; ***P < 0.001; ****P < 0.0001; ns is non-significant.

Next, the sortase-mediated coupling reaction of the nanobody to the uncaged cytokine was optimised using unmodified IL-1β-LPETGG. The initial ligation reactions were performed in 50 mM Tris/150 mM NaCl/10% glycerol pH 7.5 for 1 hour at 37 °C using equal equivalents of both IL-1β-LPETGG (19.7 kDa) and VHH-CD11c-SIINFEKL (15 kDa 4 μM each) and 0.75 equivalents of sortase A (3 μM). SDS-PAGE (Fig. S8A, ESI†) confirmed formation of the expected 35 kDa conjugate (red arrow). The gel showed additional bands at molecular weights lower then IL-1β-LPETGG or sortase A, even in the absence of the nanobody. Further research into these proteins was not performed. The assumption was made that these bands originated from hydrolysis of the bond formed between sortase A and IL-1β-LPETGG. The result is cleavage at the LPETGG site, generating a smaller fragment of IL-1β. Optimisation experiments indicated that higher sortase concentrations or prolonged reaction times (≥2 h) reduced product yield and shorter reaction times (1–15 min) improved efficiency (Fig. S8B–D, ESI†). This was most likely due to the fact that sortase A can also hydrolyse the formed bonds between the nanobody and the IL-1β-LPETGG.^89^ The most effective ligation conditions, minimizing side product formation due to sortase A–mediated hydrolysis, were 4 μM cytokine, 8 μM nanobody, and 3 μM sortase A with a 15-minute reaction time. Although the reaction was not complete, the yield was sufficient for subsequent experiments (Fig. 7).

These conditions were then used to ligate the caged cytokine to the anti-CD11c nanobody. IL-1β-LPETGG (5.0 μM) was caged using 3.5 mM NHS-TCO and the caged product was coupled to VHH-CD11c-SIINFEKL by sortase A using the optimal conditions found in Fig. S8B–D (ESI†). The red arrow indicated the 35 kDa product formed between caged IL-1β-LPETGG and VHH-CD11c-SIINFEKL, which was only formed when both substrates and sortase A were present (Fig. S9A, ESI†). The green arrow indicated an intermediate (38 kDa) formed between sortase A and caged IL-1β-LPETGG in the absence of VHH-CD11c-SIINFEKL (Fig. S9A, ESI†). Finally, the dark purple arrow indicated the presence of uncoupled caged IL-1β-LPETGG (Fig. S9A, ESI†). Product formation was assessed with IL-1β-specific western blot (Fig. S9B, ESI†) and, for the TCO-modified products, by reaction with BODIPY-TMR-tetrazine (Fig. S9A, ESI†). Tetrazine reacts with *trans*-cyclooctene (TCO), resulting in decaging of most substrates; however, fluorophores are highly sensitive, and while release is initiated, a sufficient amount of the TCO–tetrazine intermediate remains stabilized to be detected *via* gel analysis. Additionally, the steric bulk of the fluorophore can hinder release.

To optimise the deprotection step, crude mixtures of IL-1β-VHH-CD11c-SIINFEKL were tested. These were made by first caging IL-1β-LPETGG with 3.5 mM, 2.1 mM, or 0.7 mM NHS-TCO, followed by ligation to the nanobody. The resulting mixtures were added to HEK-Blue IL-1β cells to measure activity. Caged IL-1β binding to its IL-1R receptor compared to uncaged IL-1β was 2% with 3.5 mM NHS-TCO, 5% with 2.1 mM NHS-TCO, and 16% with 0.7 mM NHS-TCO (Fig. 7B, Fig. S10A and B, ESI†). Treatment with (2PyrH)_2_Tz successfully restored full activity for the 2.1 and 0.7 mM samples, but activity recovery for the 3.5 mM sample remained below 20%. Purification of the crude mixtures *via* size exclusion chromatography (SEC) yielded limited success, and needs to be further optimised (Fig. S11A–D, ESI†).

## Conclusion

We here present a general method for caging and uncaging proteins optimised by performing the caging reaction at lower temperatures. This has yielded caged variants of IL-1β, IL-2 and IFN-γ that could be uncaged. Only the window between caging and uncaging of TNF proved small (from 50% to 60% activity), likely due to its trimeric nature, making reactivation more difficult. We also showed that caged cytokines can be conjugated to uncaged nanobody through a sortase ligation reaction, leading to a construct that could be caged and decaged. If it can be targeted to an antigen, kept there, and then uncaged needs to be evaluated. This is the first step into the design of a strategy that could be of interest to generating immunocytokines with reduced systemic toxicity.

## Conflicts of interest

There are no conflicts to declare.

## Supplementary Material

CB-OLF-D5CB00113G-s001

## Data Availability

The data supporting this article have been included as part of the ESI.†

## References

[cit1] Carnemolla B., Borsi L., Balza E., Castellani P., Meazza R., Berndt A., Ferrini S., Kosmehl H., Neri D., Zardi L. (2002). Blood.

[cit2] Borsi L., Balza E., Carnemolla B., Sassi F., Castellani P., Berndt A., Kosmehl H., Biro A., Siri A., Orecchia P., Grassi J., Neri D., Zardi L. (2003). Blood.

[cit3] Boersma B., Poinot H., Pommier A. (2024). Pharmaceutics.

[cit4] Gillies S. D., Reilly E. B., Lo K. M., Reisfeld R. A. (1992). Proc. Natl. Acad. Sci. U. S. A..

[cit5] Panelli M. C., White R., Foster M., Martin B., Wang E., Smith K., Marincola F. M. (2004). J. Transl. Med..

[cit6] Jia F., Wang G., Xu J., Long J., Deng F., Jiang W. (2021). Aging.

[cit7] Danielli R., Patuzzo R., Di Giacomo A. M., Gallino G., Maurichi A., Di Florio A., Cutaia O., Lazzeri A., Fazio C., Miracco C., Giovannoni L., Elia G., Neri D., Maio M., Santinami M. (2015). Cancer Immunol. Immunother..

[cit8] Gout D. Y., Groen L. S., Van Egmond M. (2022). Cell. Mol. Life Sci..

[cit9] Gillies S. D., Lan Y., Hettmann T., Brunkhorst B., Sun Y., Mueller S. O., Lo K.-M. (2011). Clin. Cancer Res..

[cit10] Klein C., Waldhauer I., Nicolini V. G., Freimoser-Grundschober A., Nayak T., Vugts D. J., Dunn C., Bolijn M., Benz J., Stihle M., Lang S., Roemmele M., Hofer T., Van Puijenbroek E., Wittig D., Moser S., Ast O., Brünker P., Gorr I. H., Neumann S., De Vera Mudry M. C., Hinton H., Crameri F., Saro J., Evers S., Gerdes C., Bacac M., Van Dongen G., Moessner E., Umaña P. (2017). OncoImmunology.

[cit11] Waldmann T. A. (2006). Nat. Rev. Immunol..

[cit12] Wolf S. F., Temple P. A., Kobayashi M., Young D., Dicig M., Lowe L., Dzialo R., Fitz L., Ferenz C., Hewick R. M. (1991). J. Immunol..

[cit13] Gillies S. D. (2013). Protein Eng., Des. Sel..

[cit14] Hsu E. J., Cao X., Moon B., Bae J., Sun Z., Liu Z., Fu Y.-X. (2021). Nat. Commun..

[cit15] Li J., Chen P. R. (2016). Nat. Chem. Biol..

[cit16] Versteegen R. M., Rossin R., Hoeve W. T., Janssen H. M., Robillard M. S. (2013). Angew. Chem., Int. Ed..

[cit17] Li J., Jia S., Chen P. R. (2014). Nat. Chem. Biol..

[cit18] Carlson J. C. T., Mikula H., Weissleder R. (2018). J. Am. Chem. Soc..

[cit19] Rossin R., Versteegen R. M., Wu J., Khasanov A., Wessels H. J., Steenbergen E. J., Hoeve W. T., Janssen H. M., Van Onzen A. H. A. M., Hudson P. J., Robillard M. S. (2018). Nat. Commun..

[cit20] De Geus M. A. R., Groenewold G. J. M., Maurits E., Araman C., Van Kasteren S. I. (2020). Chem. Sci..

[cit21] Oliveira B. L., Guo Z., Bernardes G. J. L. (2017). Chem. Soc. Rev..

[cit22] Rossin R., Van Duijnhoven S. M. J., Hoeve W. T., Janssen H. M., Kleijn L. H. J., Hoeben F. J. M., Versteegen R. M., Robillard M. S. (2016). Bioconjugate Chem..

[cit23] Srinivasan S. , Yee N. A., Zakharian M., Alečković M., Mahmoodi A., Nguyen T.-H. and Oneto J. M. M., bioRxiv, 2023, preprint 10.1101/2023.03.28.534654

[cit24] Blackman M. L., Royzen M., Fox J. M. (2008). J. Am. Chem. Soc..

[cit25] Friederich J., Xu C., Raunft P., Fuchs H. L. S., Brönstrup M. (2023). Chem. Commun..

[cit26] Dzijak R., Galeta J., Vázquez A., Kozák J., Matoušová M., Fulka H., Dračínský M., Vrabel M. (2020). J. Am. Chem. Soc..

[cit27] Ligthart N. A. M., De Geus M. A. R., Van De Plassche M. A. T., García D. T., Isendoorn M. M. E., Reinalda L., Ofman D., Van Leeuwen T., Van Kasteren S. I. (2023). J. Am. Chem. Soc..

[cit28] Van Der Gracht A. M. F., De Geus M. a R., Camps M. G. M., Ruckwardt T. J., Sarris A. J. C., Bremmers J., Maurits E., Pawlak J. B., Posthoorn M. M., Bonger K. M., Filippov D. V., Overkleeft H. S., Robillard M. S., Ossendorp F., Van Kasteren S. I. (2018). ACS Chem. Biol..

[cit29] Zhang G., Li J., Xie R., Fan X., Liu Y., Zheng S., Ge Y., Chen P. R. (2016). ACS Cent. Sci..

[cit30] Den Brok M. H. M. G. M. , Robillard M. S., Rossin R., Kleijn L. H. J., De Roode K. E., Wouters L. M., Zijlmans L. M. and Versteegen R. M., U.S. Pat. Appl. US2025/0114489A1, 2025 10.3390/ph18091380 PMC12472795 41011249

[cit31] Zheng X., Wu Y., Bi J., Huang Y., Cheng Y., Li Y., Wu Y., Cao G., Tian Z. (2022). Cell. Mol. Immunol..

[cit32] Schwager K., Hemmerle T., Aebischer D., Neri D. (2012). J. Invest. Dermatol..

[cit33] Hemmerle T., Probst P., Giovannoni L., Green A. J., Meyer T., Neri D. (2013). Br. J. Cancer.

[cit34] Saha B., Prasanna S. J., Chandrasekar B., Nandi D. (2009). Cytokine.

[cit35] Street S. E. A., Trapani J. A., MacGregor D., Smyth M. J. (2002). J. Exp. Med..

[cit36] Mantovani A., Barajon I., Garlanda C. (2017). Immunol. Rev..

[cit37] Baker K. J., Houston A., Brint E. (2019). Front. Immunol..

[cit38] Carmi Y., Dotan S., Rider P., Kaplanov I., White M. R., Baron R., Abutbul S., Huszar M., Dinarello C. A., Apte R. N., Voronov E. (2013). J. Immunol..

[cit39] Bent R., Moll L., Grabbe S., Bros M. (2018). Int. J. Mol. Sci..

[cit40] Krelin Y., Voronov E., Dotan S., Elkabets M., Reich E., Fogel M., Huszar M., Iwakura Y., Segal S., Dinarello C. A., Apte R. N. (2007). Cancer Res..

[cit41] Allen I. C., TeKippe E. M., Woodford R.-M. T., Uronis J. M., Holl E. K., Rogers A. B., Herfarth H. H., Jobin C., Ting J. P.-Y. (2010). J. Exp. Med..

[cit42] Haabeth O. A. W., Lorvik K. B., Yagita H., Bogen B., Corthay A. (2015). OncoImmunology.

[cit43] Sims J. E. (2002). Curr. Opin. Immunol..

[cit44] Labriola-Tompkins E., Chandran C., Kaffka K. L., Biondi D., Graves B. J., Hatada M., Madison V. S., Karas J., Kilian P. L., Ju G. (1991). Proc. Natl. Acad. Sci. U. S. A..

[cit45] Sims J. E., Acres R. B., Grubin C. E., McMahan C. J., Wignall J. M., March C. J., Dower S. K. (1989). Proc. Natl. Acad. Sci. U. S. A..

[cit46] Lopez-Castejon G., Brough D. (2011). Cytokine Growth Factor Rev..

[cit47] Sims J. E., Gayle M. A., Slack J. L., Alderson M. R., Bird T. A., Giri J. G., Colotta F., Re F., Mantovani A., Shanebeck K. (1993). Proc. Natl. Acad. Sci. U. S. A..

[cit48] Joshi B. H., Puri R. K. (2004). Protein Expression Purif..

[cit49] Liegel J., Avigan D., Rosenblatt J. (2018). Expert Rev. Hematol..

[cit50] Costes V., Portier M., Lu Z., Rossi J., Bataille R., Klein B. (1998). Br. J. Haematol..

[cit51] North R. J., Neubauer R. H., Huang J. J., Newton R. C., Loveless S. E. (1988). J. Exp. Med..

[cit52] Hess C., Neri D. (2014). Protein Eng., Des. Sel..

[cit53] Uptima, TCO (Trans-CycloOctyne) Reagents for “Click Chemistry” – Amine Reactive Products Information, https://www.interchim.fr/ft/M/MRU990.pdf, accessed March 16, 2023

[cit54] Van Oostrum J., Priestle J. P., Grutter M. G., Schmitz A. (1991). J. Struct. Biol..

[cit55] InvivoGen, RAW-Blue™ cells, https://www.invivogen.com/raw-blue, accessed March 17, 2023

[cit56] InvivoGen, HEK-Blue™ IL-1β cells, https://www.invivogen.com/hek-blue-il1b, accessed March 17, 2023

[cit57] Merck, Colorimetric alkaline phosphatase and peroxidase substrate detection systems, https://www.sigmaaldrich.com/NL/en/technical-documents/technical-article/protein-biology/immunohistochemistry/colorimetric-alkaline, accessed March 16, 2023

[cit58] InvivoGen, QUANTI-Blue™ solution, 2020, https://www.invivogen.com/quanti-blue, accessed March 17, 2023

[cit59] Liu T., Zhang L., Joo D., Sun S.-C. (2017). Signal Transduction Targeted Ther..

[cit60] Casadio R., Frigimelica E., Bossù P., Neumann D., Martin M. U., Tagliabue A., Boraschi D. (2001). FEBS Lett..

[cit61] Ye C., Brand D., Zheng S. G. (2018). Sig. Transduct. Targeted Ther..

[cit62] Josephson K., Logsdon N. J., Walter M. R. (2001). Immunity.

[cit63] Baeyens K. J., De Bondt H. L., Raeymaekers A., Fiers W., De Ranter C. J. (1999). J. Struct. Biol..

[cit64] Wilkovitsch M., Kuba W., Keppel P., Sohr B., Löffler A., Kronister S., Del Castillo A. F., Goldeck M., Dzijak R., Rahm M., Vrabel M., Svatunek D., Carlson J., Mikula H. (2024). Angew. Chem., Int. Ed..

[cit65] Fan X., Ge Y., Lin F., Yang Y., Zhang G., Ngai W. S. C., Lin Z., Zheng S., Wang J., Zhao J., Li J., Chen P. R. (2016). Angew. Chem., Int. Ed..

[cit66] Policarpo R. L., Kang H., Liao X., Rabideau A. E., Simon M. D., Pentelute B. L. (2014). Angew. Chem., Int. Ed..

[cit67] Levary D. A., Parthasarathy R., Boder E. T., Ackerman M. E. (2011). PLoS One.

[cit68] Li J., Zhang Y., Soubias O., Khago D., Chao F.-A., Li Y., Shaw K., Byrd R. A. (2020). J. Biol. Chem..

[cit69] Carnemolla B., Borsi L., Balza E., Castellani P., Meazza R., Berndt A., Ferrini S., Kosmehl H., Neri D., Zardi L. (2002). Blood.

[cit70] Borsi L., Balza E., Carnemolla B., Sassi F., Castellani P., Berndt A., Kosmehl H., Biro A., Siri A., Orecchia P., Grassi J., Neri D., Zardi L. (2003). Blood.

[cit71] Asaadi Y., Jouneghani F. F., Janani S., Rahbarizadeh F. (2021). Biomark. Res..

[cit72] Catania C., Maur M., Berardi R., Rocca A., Di Giacomo A. M., Spitaleri G., Masini C., Pierantoni C., González-Iglesias R., Zigon G., Tasciotti A., Giovannoni L., Lovato V., Elia G., Menssen H. D., Neri D., Cascinu S., Conte P. F., De Braud F. (2015). Cell Adhes. Migr..

[cit73] Schwager K., Kaspar M., Bootz F., Marcolongo R., Paresce E., Neri D., Trachsel E. (2009). Arthritis Res. Ther..

[cit74] Gafner V., Trachsel E., Neri D. (2006). Int. J. Cancer.

[cit75] Pasche N., Woytschak J., Wulhfard S., Villa A., Frey K., Neri D. (2011). J. Biotechnol..

[cit76] Heiss J., Grün K., Tempel L., Matasci M., Schrepper A., Schwarzer M., Bauer R., Förster M., Berndt A., Jung C., Schulze P. C., Neri D., Franz M. (2022). Eur. J. Clin. Invest..

[cit77] Ebbinghaus C., Ronca R., Kaspar M., Grabulovski D., Berndt A., Kosmehl H., Zardi L., Neri D. (2005). Int. J. Cancer.

[cit78] Bathula N. V., Bommadevara H., Hayes J. M. (2020). Cancer Biother. Radiopharm..

[cit79] Muyldermans S. (2013). Annu. Rev. Biochem..

[cit80] Mosley B., Urdal D. L., Prickett K. S., Larsen A., Cosman D., Conlon P. J., Gillis S., Dower S. K. (1987). J. Biol. Chem..

[cit81] Van Rosmalen M., Krom M., Merkx M. (2017). Biochemistry.

[cit82] Heck T., Pham P.-H., Yerlikaya A., Thöny-Meyer L., Richter M. (2014). Catal. Sci. Technol..

[cit83] Beerli R. R., Hell T., Merkel A. S., Grawunder U. (2015). PLoS One.

[cit84] Popp M. W., Antos J. M., Ploegh H. L. (2009). Curr. Protoc. Protein Sci..

[cit85] Agilent, ArcticExpress RIL Competent Cells and ArcticExpress RP Competent Cells Instruction Manual, 2010

[cit86] Ferrer M., Chernikova T. N., Yakimov M. M., Golyshin P. N., Timmis K. N. (2003). Nat. Biotechnol..

[cit87] Studier F. W., Rosenberg A. H., Dunn J. J., Dubendorff J. W. (1970). Methods Enzymol..

[cit88] Gall C. M. L., Weiden J., Eggermont L. J., Figdor C. G. (2018). Nat. Mater..

[cit89] Huang X., Aulabaugh A., Ding W., Kapoor B., Alksne L., Tabei K., Ellestad G. (2003). Biochemistry.

