# Supplementary material for: trans-Cyclooctene-caged-IL-1β immunocytokine-constructs ligated to unmodified nanobodies allow click-2-release-based control of cytokine activity
Source: RSC Chem Biol. 2025 Jun 4;6(7):1068–78. doi: 10.1039/d5cb00113g (PMC12151080; doi:10.1039/d5cb00113g)
Supplement: CB-006-D5CB00113G-s001 [file CB-006-D5CB00113G-s001.pdf]

Supplementary Information (SI) for RSC Chemical Biology.
This journal is © The Royal Society of Chemistry 2025

Supplementary Figures

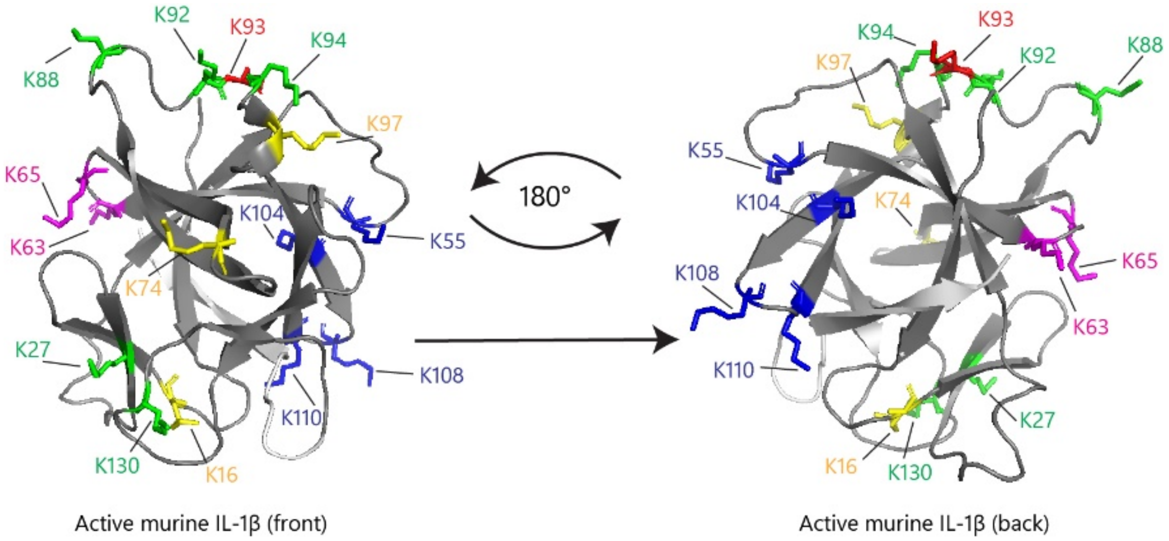

Figure S1 Crystal structure of murine IL-1β with highlighted lysine-residues. *Murine IL-1β contains 15 lysine residues which are highlighted according to functionality: green for essential in receptor binding; red for receptor binding and hydrophilic interactions; magenta for IL-1RAcP interacting residues; blue for residues in close proximity of receptor interacting residues; yellow for the remaining residues. Crystal structure was modified based on Oostrum et al. (PDB: 2MIB).[54]*

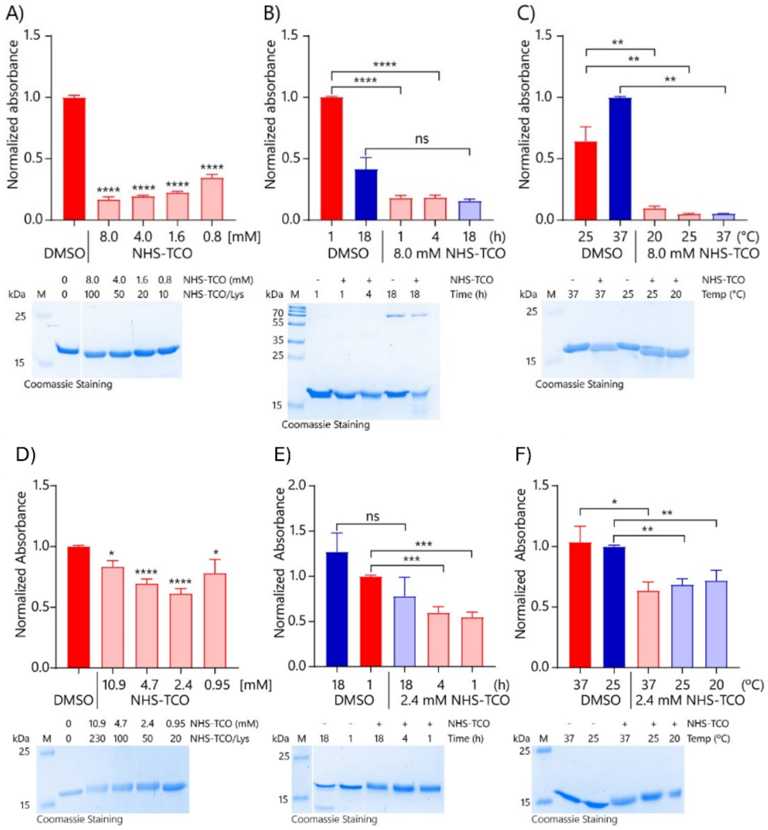

Figure S2 Optimisation of caging reaction on IL-1β and TNF-α analysed using ELISA. *The caging reaction using NHS-TCO was optimised for A) IL-1β with concentration NHS-TCO (N=3); B) IL-1β with caging time (h) (N=5); and C) IL-1β with caging temperature (°C) (N=3). The initial standard caging conditions were set at caging with 8 mM NHS-TCO (B,C) for 1 hour (A,C) at 37°C (A,B). D) TNF-α with concentration of NHS-TCO (N=3), E) TNF-α caging for various periods of time (h) (N=3) and F) TNF-α caging at various temperatures (°C) (N=4). The initial standard*

*caging conditions were set at caging with 2.4 mM NHS-TCO (E,F) for 1 hour (D,F) at 37°C (D,E). Caging was compared with the DMSO control which gave the maximum signal. Bright colours indicate DMSO only samples and pastels the TCO-treated samples. Colour groups indicate different conditions with respective DMSO controls. Equal protein application was confirmed using SDS-PAGE coomassie staining (below each graph). Data were plotted as mean signal ± SEM. Significances are indicated as follows: * P<0.05; ** P<0.01; *** P< 0.001; **** P<0.0001; ns is non-significant.*

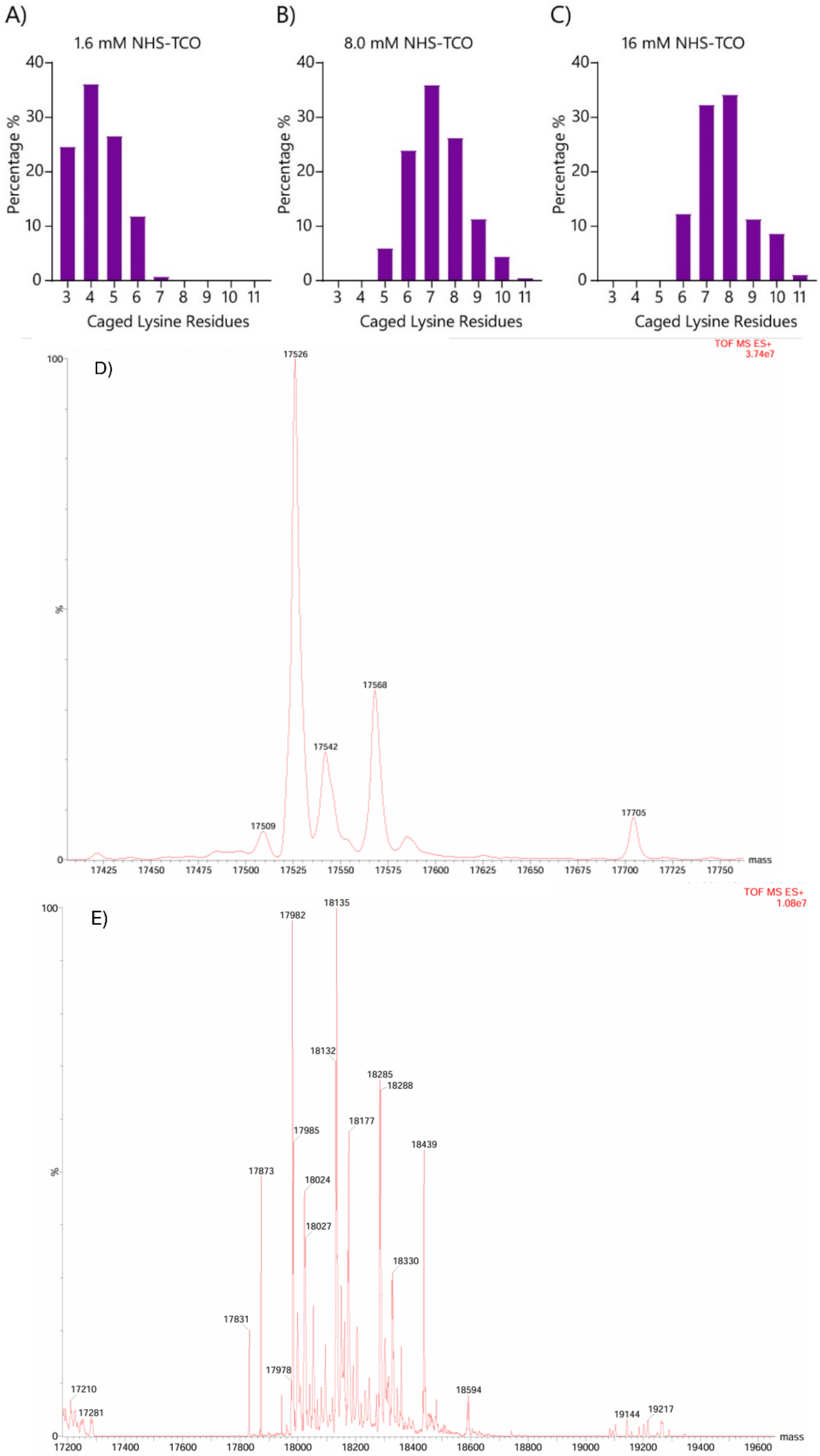

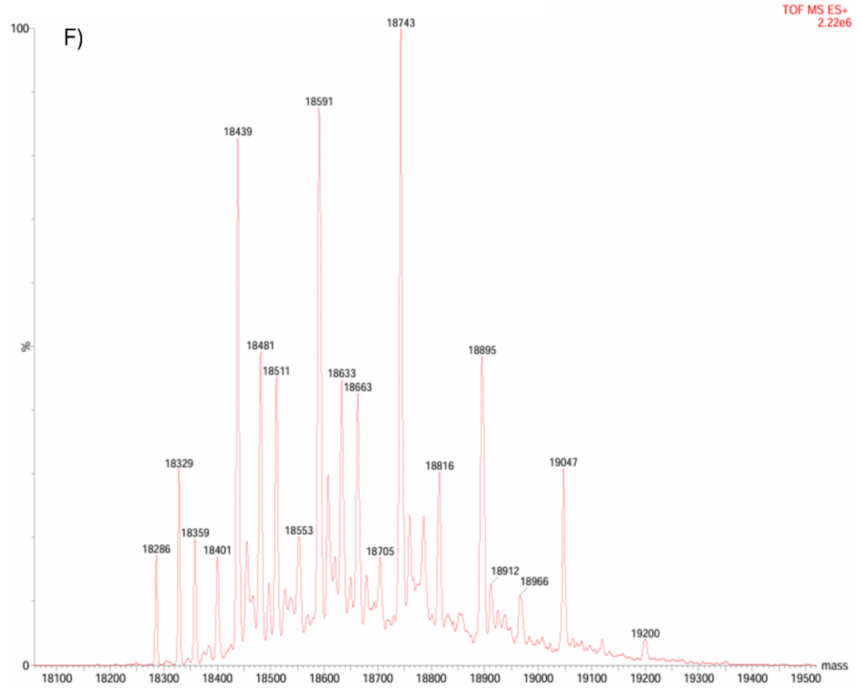

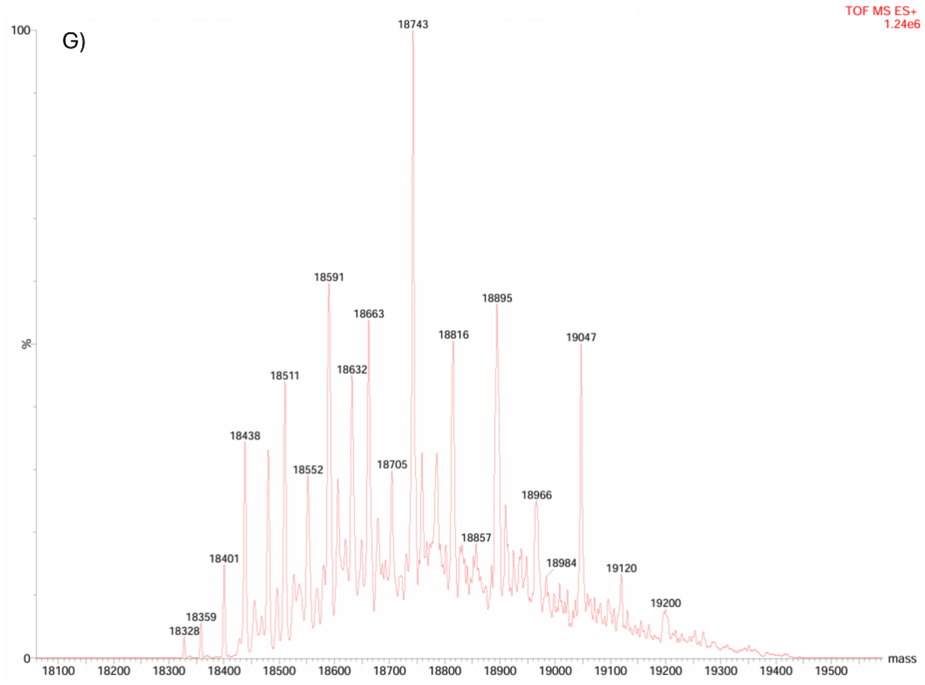

Figure S3 Distribution amount of caged lysines in IL-1β, caged with different amounts of NHS-TCO analysed by ESI LC-MS. *The amount of caged lysine residues of IL-1β (11 μM) treated with A) 1.6 mM, B) 8.0 mM and C) 16 mM NHS-TCO determined using ESI LC-MS. D-G shows raw Mass spectrometry data of IL-1β (11 μM) treated with D) 0 mM (control spectrum, peak at m/z 17526 corresponds to the unmodified protein, E) 1.6 mM, F) 8.0 mM and G) 16 mM NHS-TCO. IL-1β was caged in 20 mM HEPES for 1 hour at 37°C, following buffer exchange using Zeba^{TM} Spin Desalting columns to 10 mM ammonium acetate pH 6. In general the more NHS-TCO was added, the more caged lysines were present. These are normalised data to the total amount of lysines present.*

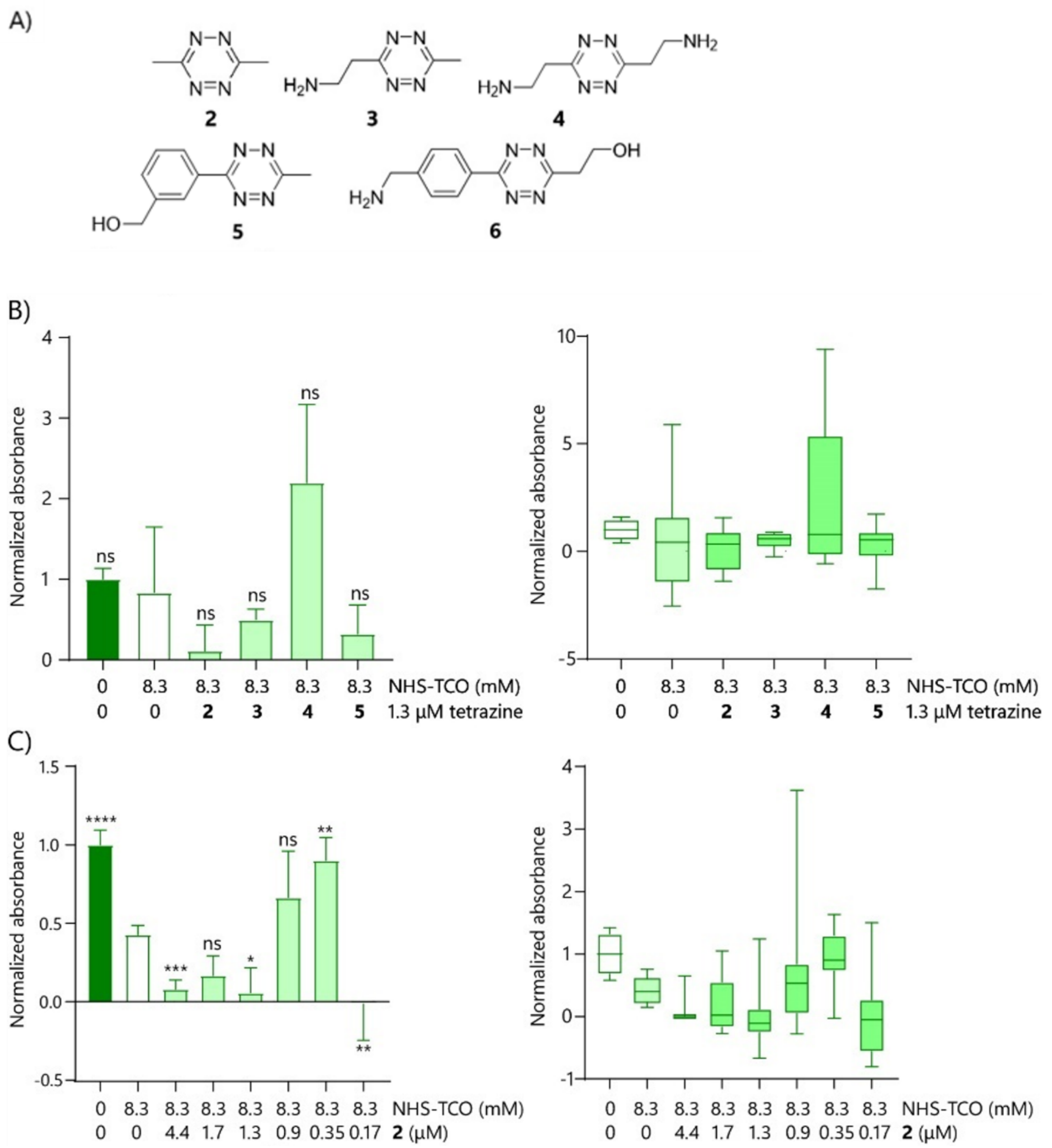

Figure S4 Initial decaging optimisation of IL-1β and IL-1β-LPETGG using different tetrazines analysed by RAW-Blue cells and HEK-Blue IL-1β cells, respectively *A) Various tetrazines tested for decaging of IL-1β (B,C) and IL-1β-LPETGG (D). B,C) IL-1β (11 µM) was caged with 8.3 mM NHS-TCO for 1 hour at 37°C in 20 mM HEPES pH 8, dialysed in 20 mM HEPES pH 7 to remove unreacted NHS-TCO following addition to RAW-Blue cells in the applicable concentration. Decaging was performed with B) 1.3 µM of different tetrazines* ***2-6*** *(N=6), or C) different concentrations of 2 (DMT) (N=6), and took place for 18-20 hours at 37°C on the cells. Decaging was performed with 25 µM of different tetrazines and took place for 18-20 hours at 37°C on the cells (N=6). Bright colours indicate DMSO only samples and pastels the tetrazine treated samples. Data were plotted as mean signal ± SEM. Significances are indicated as follows: * P<0.05; ** P<0.01; *** P<0.001; **** P<0.0001; ns is non-significant.*

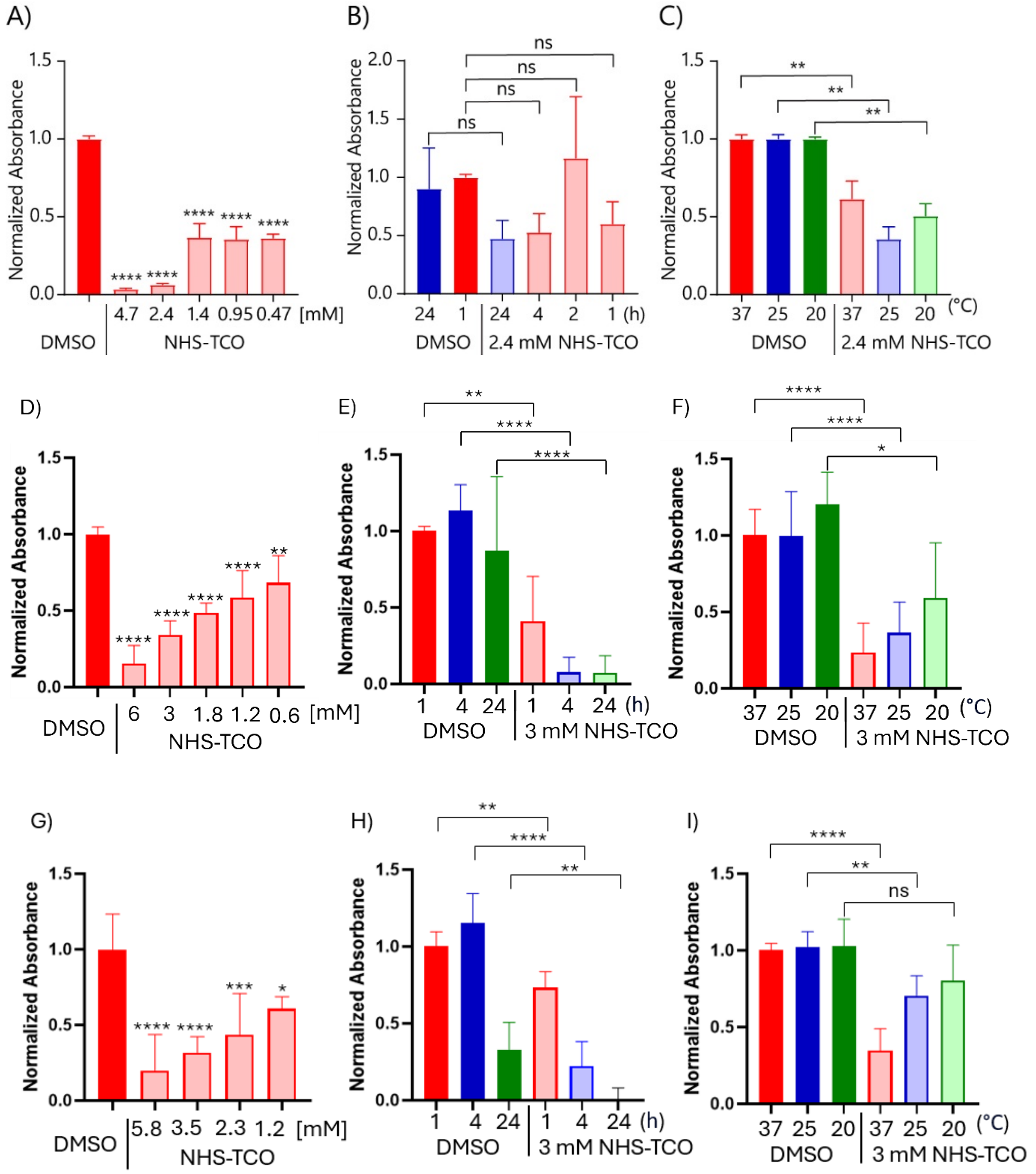

Figure S5 Analysis of caging optimisation of TNF-α and IL-2 on HEK-Blue cells using QUANTI-Blue colorimetric assay and IFN-γ on THP-1-DUAL cells using QUANTI-Luc. *A-C Optimalisation of TNF-α-6His (0.1 mg/mL or 5.4 µM) caging in 20 mM HEPES pH 8 was assessed with stimulated HEK-Blue TNF-α cells. A) Analyses of different concentrations of NHS-TCO (N=5), B) for various periods of time (h) (N=3) and C) at various temperatures (°C) (N=3) were performed. D-F Optimalisation of IL-2 (0.1 mg/mL or 5.6 µM) caging in 20 mM HEPES pH 8 was assessed with stimulated HEK-Blue IL-2 cells. D) Analyses of different concentrations of NHS-TCO (N=4), E) for various periods of time (h) (N=3) and F) at various temperatures (°C) (N=5) were performed. G-I Optimalisation of IFN-γ (0.1 mg/mL or 5.9 µM) caging in PBS was assessed with stimulated THP-1-DUAL cells. G) Analyses of different concentrations of NHS-TCO (N=4), H) for various periods of time (h) (N=3) and I) at various temperatures (°C) (N=3) were performed. Caging was compared with the DMSO control which gave the maximum signal. Bright colours indicate DMSO only samples and pastels the TCO-treated samples. Colour groups indicate different conditions with respective DMSO controls. In general, caging reduced the signal compared to the DMSO control.*

*Data were plotted as mean signal ± SEM. Significances are indicated as follows: * P<0.05; ** P<0.01; *** P<0.001; **** P<0.0001; ns is non-significant.*

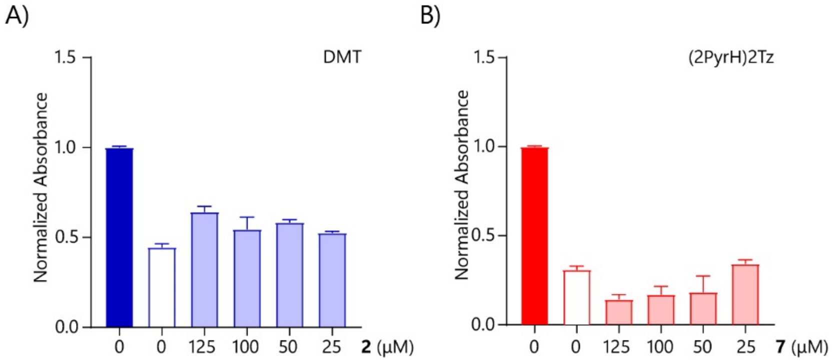

Figure S6 Decaging optimisation of TNF-α-LPETGG-6His analysed by HEK-Blue TNF-α*. TNF-α-LPETGG-6His (0.1 mg/mL or 5.1 µM) in 20 mM HEPES, caged with 0.47 mM NHS-TCO for 24 hours at 10°C, was decaged with A) increasing concentrations of DMT (N=2) or B) increasing concentrations of $(2PyrH)_2Tz$ (N=2) on HEK-Blue TNF-α cells. Decaging took place for 20-24 hours at 37°C. Tetrazine was supplied in 0.5 µL DMSO. Bright colours indicate DMSO only samples and pastels the tetrazine treated samples. In general, these preliminary data showed that increasing concentration DMT caused more decaging, while decaging with (2PyrH)2Tz did not result in decaging. Data were plotted as mean signal ± SEM. Due to the limited number of datapoints no significance could be determined.*

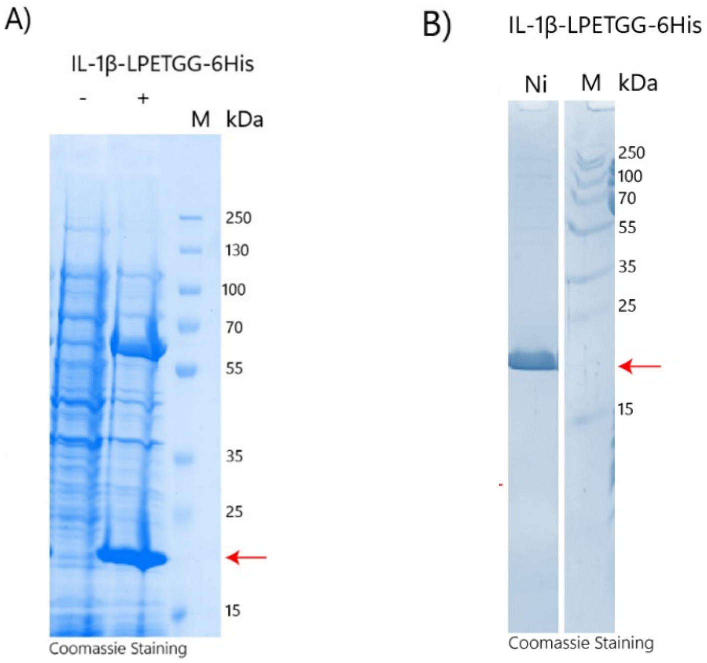

Figure S7 Bacterial expression of IL-1β-LPETGG upon IPTG induction. A) *SDS-PAGE analysis of IPTG induced expression of IL-1β-LPETGG-6His (~19 kDa) by E.coli ArcticExpress (DE3) RP B) Purification of IL-1β-LPETGG-6His using nickel column purification (Ni), analysed by SDS-PAGE. The expressed proteins are highlighted by the red arrows at the respective gels.*

.

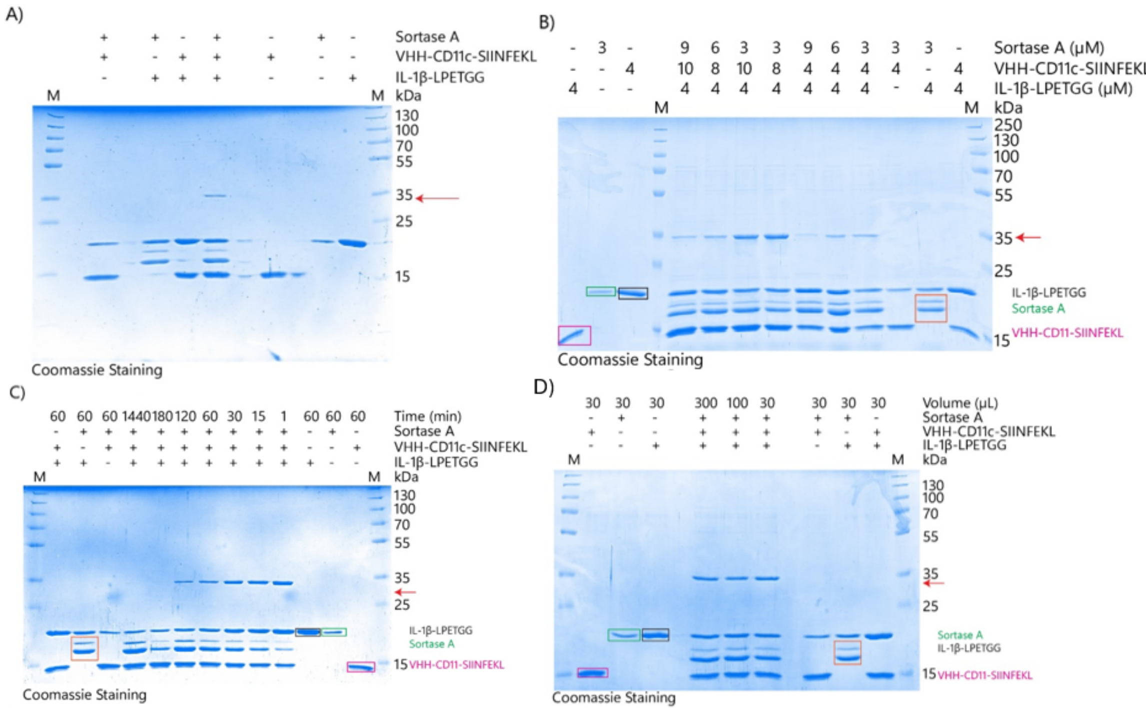

Figure S8 Sortase reaction with IL-1β-LPETGG and VHH-CD11c-SIINFEKL analysed by SDS-PAGE
Sortase A recognises the C-terminal LPETGG-sequence of IL-1β, cleaves between the tyrosine and the glycine and couples it to the N-terminal poly-glycine tail of the VHH-CD11c-SIINFEKL nanobody. A) SDS-PAGE analysis with coomassie staining shows a product of 35 kDa (red arrow). B-C Sortase A ((19 kDa, green box) reaction with IL-1β-LPETGG (19.7 kDa, black box) and VHH-CD11c-SIINFEKL (15 kDa, pink box) was optimised and analysed using SDS-PAGE. The orange box circles the hydrolysed products from IL-1β reacting with Sortase A. The reaction was performed in 50 mM Tris pH 7.5/150 mM NaCl/10% (v/v) glycerol supplemented with 1 mM $CaCl_2$. B) Component concentrations and ratios, C) incubation time of the reaction and D) reaction volume were analysed. Using 2-3 times more VHH-CD11c-SIINFEKL compared to IL-1β (B) resulted in increased product formation (red arrows). Decreasing the incubation time to 1-15 minutes (C), increasing reaction volume up until 300 µL (D) resulted in increased product formation.

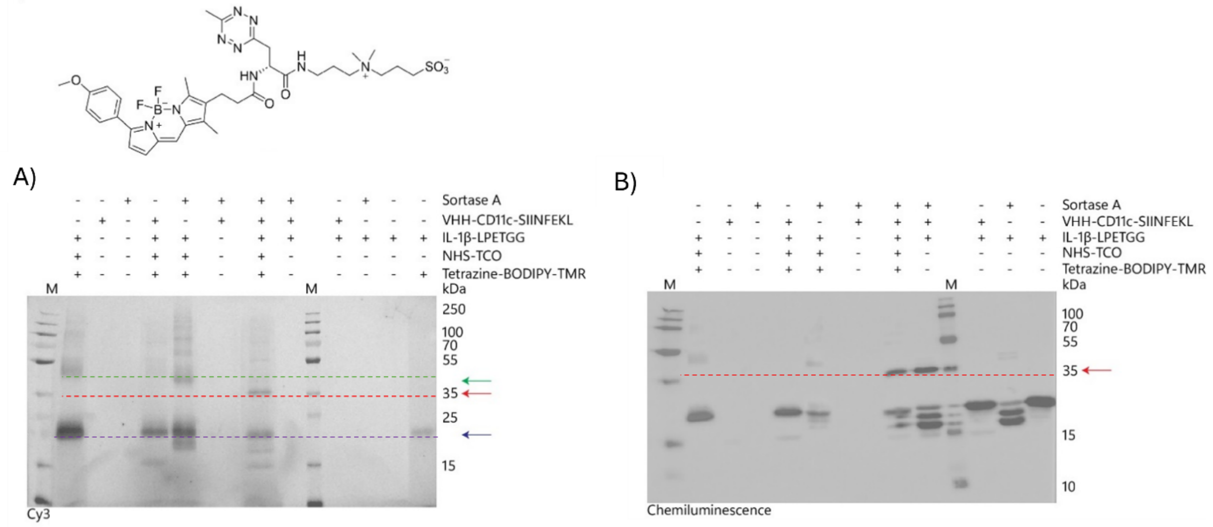

Figure S9 Sortase reaction with cagedIL-1β-LPETGG and VHH-CD11c-SIINFEKL *Sortase A recognizes the C-terminal LPETGG-sequence of IL-1β, cleaves between the tyrosine and the glycine and couples it to the N-terminal poly-glycine tail of the VHH-CD11c-SIINFEKL nanobody. IL-1β-LPETGG (5.0 µM) was first caged using 3.5 mM NHS-TCO and then coupled to VHH-CD11c-SIINFEKL using sortase A. A) Tetrazine-BODIPY-TMR was used for visualisation of caged IL-1β-LPETGG. Non-coupled IL-1β-LPETGG was indicated with the purple arrow, sortase-coupled IL-1β-LPETGG was indicated with a green arrow and product formation was indicated with the red arrow.*

B) Western blot using an IL-1β-specific antibody shows that coupling between IL-1β-LPETGG and VHH-CD11c-SIINFEKL took place (red arrows).

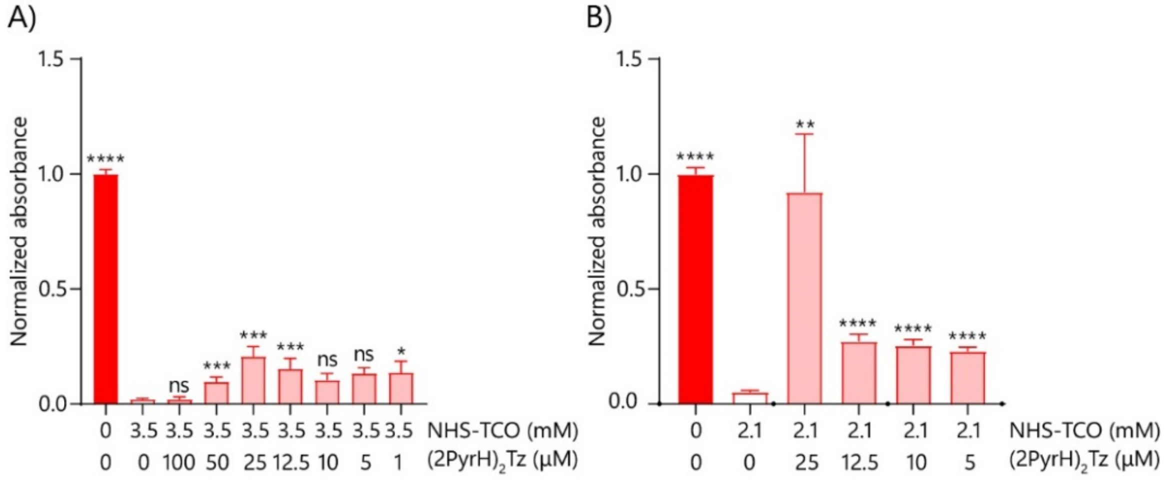

Figure S10 - Decaging optimisation of IL-1β-VHH-CD11c-SIINFEKL using HEK-Blue IL-1β cells. *The reactivation of IL-1β-VHH-CD11c-SIINFEKL (5.0 µM), caged under various conditions, by $(2PyrH)_2$ Tz was analysed on HEK-Blue IL-1β cells. Caging was performed before coupling to VHH-CD11c-SIINFEKL, in 20 mM HEPES pH 8 for A) protection with 3.5 mM NHS-TCO (for 1 hour at 37°C) (N=3)**;** B) 2.1 mM NHS-TCO (for 1 hour at 37°C) (N=5); Bright colours indicate DMSO only samples and pastels the tetrazine treated samples. Data were plotted as mean signal ± SEM. Significances are indicated as follows: * P<0.05; ** P<0.01; *** P<0.001; **** P<0.0001; ns is non-significant.*

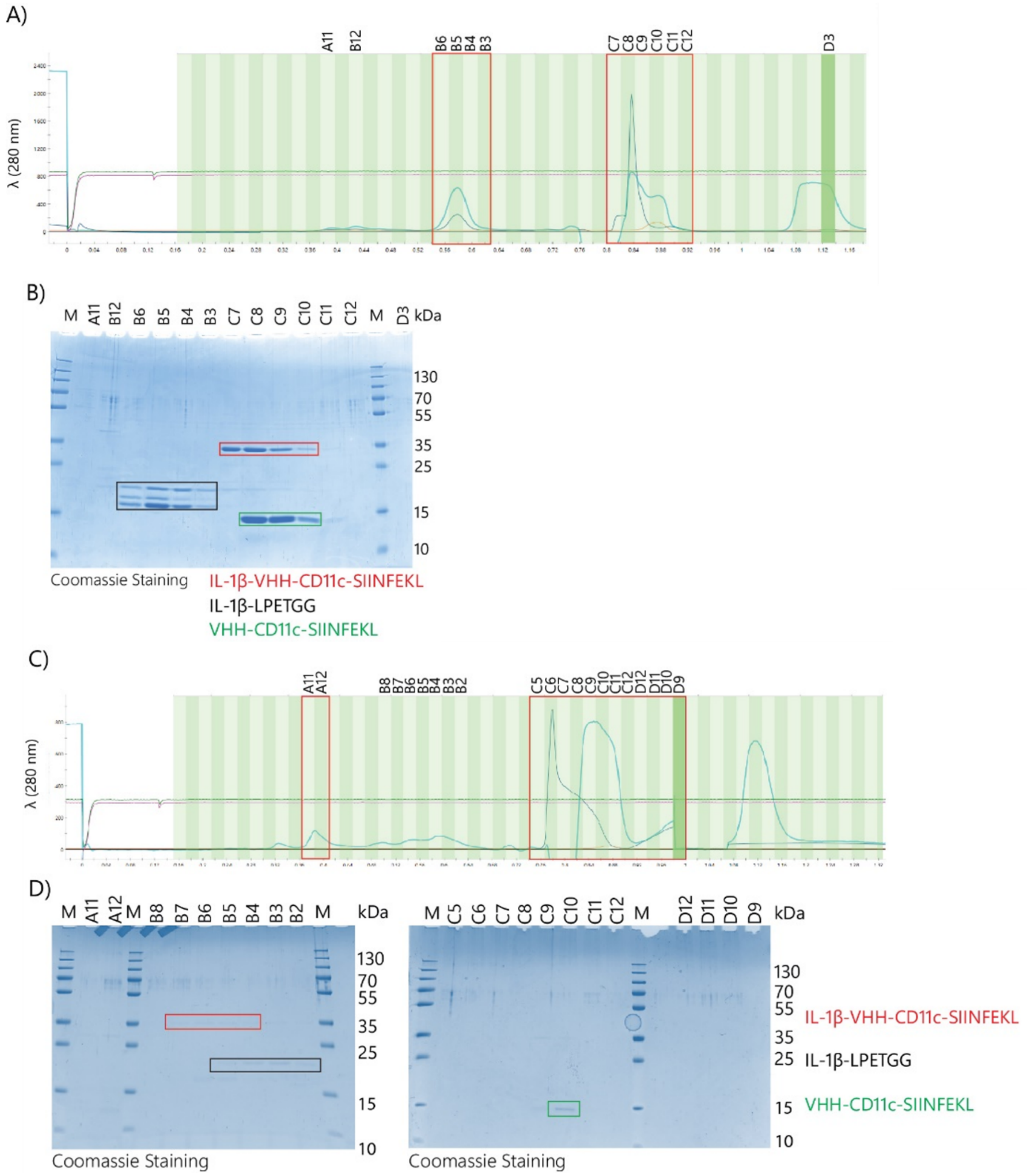

Figure S11 Initial purification non-caged and caged IL-1β-LPETGG coupled to VHH-CD11c-SIINFEKL. *A,B) Non-caged and C,D) caged IL-1β-LPETGG was coupled to VHH-CD11c-SIINFEKL and purified on a SuperdexTM 75 10/300 column equilibrated with 20 mM HEPES pH 8. A) Fractions of purification of non-caged IL-1β-VHH-CD11c-SIINFEKL were analysed on SDS-PAGE (B). C) Fractions of purification of caged IL-1β-VHH-CD11c-SIINFEKL (3.5 mM NHS-TCO) were analysed on SDS-PAGE (D). In the SDS-PAGEs, the red boxes indicate formed product, the green boxes indicate unreacted VHH-CD11c-SIINFEKL and the black boxes indicate unreacted IL-1β-LPETGG. In general, non-caged protein is more easily purified at a high concentration (>0.5 mg/mL). Purification of the caged coupled IL-1β-VHH resulted in 41 µg/mL in 500 µL volume, which is barely visible on the SDS*

Experimental Design

General reagents

All donor vectors (unless mentioned differently) were purchased from GenScript and primers were ordered at Sigma-Aldrich. All restriction enzymes, polymerases and ligases were purchased from ThermoFisher Scientific. Benzonase was purchased from Santa Cruz Biotechnology. Sodium deoxycholate monohydrate (CAS:302-95-4) and phenylmethanesulfonyl fluoride (PMSF,CAS:329-98-6) were purchased from Sigma-Aldrich. Rabbit anti-6*His (Item No. 600-401-392) was purchased from Rockland and mouse anti-rabbit IgG-HRP (Catalog # sc-2357) was purchased from Santa Cruz Biotechnology. Medium, L-glutamine, streptomycin and penicillin were purchased from Sigma-Aldrich. QUANTI-Blue (#rep-qbs), Quanti-Luc (#repqlc4lg1) and zeocin were purchased from InvivoGen.

Western blot

Denaturing acrylamide gels (SDS-PAGEs) were blotted onto 0.2 µm PVDF membranes (Trans-Blot Turbo Transfer Pack, midi format, single application of Bio-Rad) using the Turbo Trans Blot System (Bio-Rad). Blots were developed using luminol solution (25% (w/v) luminol in 0.1 M Tris pH 8.8), 100x diluted enhancer (1.1 mg/mL p-coumaric acid in DMSO) and H_2O_2.

Cytokine specific blot

Blots were washed with PBS and PBST (1x PBS pH 7.4, 0.5% Tween-80). Blocking was performed with 0.2% BSA in PBST. The primary antibody used was biotin-conjugate anti-mouse IL-1 beta (1:1000, #88-7013-88, ThermoFisher Scientific). Strep-Tactin® HRP conjugate (1:100000, #2-1502-001, IBA solutions) was used as a secondary antibody.

Blots were imaged with the ChemiDoc™ MP Imaging System of Bio-Rad with setting chemiluminescence, Cy3 and Cy5 and analysed using ImageLab Software version 4.1.

Caging and decaging procedure

Caging

IL-1β, TNF-α and IL-2 caging were performed in 20 mM HEPES pH 8 and for IFN-γ in PBS all at 10°C, 800 rpm for 24 hours. For standard conditions an excess of 10 molar equivalents 2-AX-NHS-TCO (kindly provided by Mark de Geus) was used per lysine in the cytokines. For IL-1β this equals a TCO concentration of 0.7 mM with an IL-1β concentration of 5 µM (0.1 mg/mL). For TNF-α this equals a TCO concentration of 0.45 mM with a TNF-α concentration of 5.7 µM (0.1 mg/mL). For IL-2 this equals TCO concentration of 0.6 mM with an IL-2 concentration of 5.6 µM (0.1 mg/mL). For IFN-γ this equals TCO concentration of 1.15 µM and an IFN-γ concentration of 5.9 µM (0.1 mg/mL).

Protein concentrations were determined using the Qubit™ Protein assay kit on the Qubit® 2.0 Fluorometer of Invitrogen (Life Technologies).

For IL-1β caging was visualised by using 0.6 µM Tetrazine Bodipy-TMR Sol2 incubating for 30 minutes at 25°C, 800 rpm. Gels were analysed using ChemiDoc™ MP Imaging System of Bio-Rad and ImageLab Software version 4.1.

Decaging – ELISA

Decaging was performed in 20 mM HEPES pH 8 with 2.5 mM $(2PyrH)_2Tz$ and 5.7 µM IL-1β/5.7 µM TNF-α caged with various amounts of NHS-TCO. Decaging was performed at 37°C, 300 rpm for 1 hour.

Decaging

Decaging was performed in complete medium in well with different concentrations of tetrazines (most used 25 µM $(2PyrH)_2Tz$ (kindly provided by Dr. Hannes Mikula) on cells at 37°C overnight.

ESI LC-MS experiments

Liquid chromatography-mass spectrometry analysis (LC-MS) was performed to study the caged lysine residues. Instruments Nanoacquity UPLC system (Waters) and Synapt G2Si mass spectrometer (Waters) were used and operated with Masslynx for acquisition and Ent3 software for polymer envelope signal deconvolution. Acquity UPLC M-class 300 µm x 50 mm column (Waters), packed with BEH C4 material of 1.7 µm diameter and 300 Å pore size particles was used as stationary phase. All materials used for mobile phase were of ULC/MS grade quality and were purchased from Biosolve or Sigma Aldrich. Mobile phase A was 0.1% formic acid (FA) in ultrapure water and mobile phase B was 0.1% formic acid in acetonitrile (ACN). Caged samples (described above) were filtered using Zeba™ Spin Desalting columns, 7K MWCO, 0.5 mL (ThermoFischer Scientific) equilibrated first three times with 10 mM ammonium acetate pH 6 (1 minute at 1500*g*) before loading the caged sample (2 minutes at 1500*g*). Approximately 5 µL of the sample was injected on the column followed by elution with a 10-90% mobile phase B gradient within 20 minutes ending with 10 minutes equilibration to 10% B at a flow of 2 µL/min. Ionization was executed with electro-spray ionization (ESI) via Nano-spray source with ESI emitters (New Objectives) fused silica tubing 360 µm OD x 25 µm ID tapered to 5 ± 0.5 µm (5 nL/cm void volume). Source temperature of 80°C, capillary voltage of 4.5 kV, nano flow gas of 0.25 bar, purge gas of 250 L/h, trap gas flow of 2.0 mL/min, cone gas of 100 L/h , sampling cone of 25 V, source offset of 25 , trap CE of 32 V, scan time of 3.0 sec, mass range of 400-2400 m/z settings were using in positive mode. Lock mass acquiring was done with a mixture of Leu Enk (556.2771) and Glu Fib (785.84265), lockspray voltage of 3.5 kV, Glufib fragmentation was used as calibrant. Charge state envelopes were analysed and deconvulted with the MaxEnt 1 software. LC-MS analysis was performed with help of Dr. B. I. Florea. Only the amount of cages present could be determined, the exact location of each cage remained unknown.

ELISA assay

Uncoated ELISA kit for IL-1β (#88-7013-88) and for mouse TNF-α (#88-7324-88) were used from Invitrogen. The assays were performed using 96-wells microplate half area high binding (#675061, Greiner Bio-one). Assay was performed according to the manufactures protocol with the following adaptations. Both antibodies and streptavidin-HRP were applied in 25 µL per well and blocking was performed with 100 µL per well. Samples were applied in 30 µL (80-1000 pg/mL for IL-1β or IL-1β-LPETGG and 80-10000 pg/mL for TNF-α) per well. Final substrate was supplied in 25 µL per well and the enzymatic reaction was stopped with 12.5 µL per well. Absorbance at 450 nm was measured with the Bio-Rad iMark™ Microplate Reader.

Cell culture

RAW-Blue

RAW-Blue™ (cat. raw-sp, InvivoGen) were maintained in Dulbecco's Modified Eagle's medium – high glucose (DMEM-high glucose, #D6546-500ML) supplemented with 2 mM L-glutamine, 10% (v/v) heat inactivated fetal calf serum, 100 IU/mL penicillin and 50 µg/mL streptomycin at 37°C, 5% CO_2. Every other passage (300 000 cells/mL in T75) 200 µg/mL zeocin was added for selection.

HEK-Blue IL-1β, TNF-α and IL-2

HEK-BLUE™ IL-1β (cat. Hkb-il1bv2, InvivoGen), HEK-BLUE™ TNF-α (cat. Hkb-tnfdmyd, InvivoGen) and *HEK-Blue IL-2* (cat. Hkb-il1bv2 and Hkb-il2, InvivoGen) were maintained in Dulbecco's Modified Eagle's medium – high glucose (DMEM-high glucose, #D6546-500ML) supplemented with 2 mM L-glutamine, 10% (v/v) heat inactivated fetal calf serum, 100 IU/mL penicillin and 50 µg/mL streptomycin at 37°C, 5% CO_2. Every other passage (130 000 cells/mL in T75) 200 µg/mL zeocin was added for selection.

THP-1-DUAL

THP-1-DUAL cells (cat. thpd-nfis, InvivoGen) were cultured at 37°C, 5% CO_2. Growth Medium was RPMI 1640 supplemented with 2 mM L-glutamine, 25 mM HEPES, 10% heat-inactivated fetal calf serum, 100 U/mL Pen-Strep and 100 µg/mL normocin. Every other passage 10 µg/mL blasticidin and 100 µg/mL zeocin were added. The test medium did not contain normocin, blasticidin and zeocin.

RAW-Blue assay

RAW-Blue cells were seeded at 50 000 cells/well in a 96-wells flat bottom plate, in growth medium without zeocin and incubated for 18-24 hours at 37°C, 5% CO_2. Samples were prepared 10 times concentrated in a separate 96-wells plate. The medium of the cells was replaced with 90 µL fresh medium and 10 µL sample reaching a final cytokine concentration of 15-1000 ng/mL. Cells were incubated for 18-20 hours at 37°C, 5% CO_2, centrifuged (2 minutes, 1000*g* at room temperature) and the 20 µL supernatant was incubated with 180 µL QUANTI-Blue in a new 96-wells plate at 37°C for 30 minutes to 6 hours. Absorbance at 655 nm was measured using the Bio-Rad iMARK Microplate reader.

HEK-BLUE IL-1β assay

Protein samples were prepared 4 times concentrated in a separate 96-wells plate of which 50 µL per well was transferred to the assay plate. HEK-BLUE IL-1β cells were seeded at 45 000 cells/well in 150 µL medium. The final protein concentration ranged 15.6 – 1000 ng/mL. Cells were incubated for 18-20 hours at 37°C, 5% CO_2 whereafter 20 µL supernatant was added to 180 µL QUANTI-Blue in a new 96-wells plate and incubated at 37°C for 30 minutes – 3 hours. Absorbance at 655 nm was measured using the Bio-Rad iMARK Microplate reader.

HEK-Blue TNF-α assay

Protein samples were prepared 10 times concentrated in a separate 96-wells plate of which 20 µL per well was transferred to the assay plate. HEK-BLUE TNF-α cells were seeded at 50 000 cells/well in 180 µL medium. The final protein concentration ranged 78 – 5000 pg/mL. Cells were incubated for 18-20 hours at 37°C, 5% CO_2 whereafter 20 µL medium was added to 180 µL QUANTI-Blue in a new 96-wells plate and incubated at 37°C for 30 minutes – 3 hours. Absorbance at 655 nm was measured using the Bio-Rad iMARK Microplate reader.

HEK-BLUE IL-2 assay

Protein samples were prepared 62.5 times concentrated in a separate 96-well plate, of which 20 µL per well was transferred to the assay plate. HEK-BLUE IL-2 cells were seeded at 50. cells/well in 180 µL medium. The final IL-2 concentration ranged from 2.5 to 160 ng/mL (0.14-9 nM). Cells were incubated for 20–24 hours at 37°C, 5% CO_2. Thereafter, 20 µL of each well was added to 180 µL QUANTI-Blue (#rep-qbs, InvivoGen) in a new 96-well plate and incubated at 37°C. Absorbance at 655 nm was measured every 30 minutes for 2 hours, with a final readout at 3 hours, using the Bio-Rad iMARK Microplate reader.

THP1-DUAL assay

Protein samples were prepared 25 times concentrated in a separate 96-well plate, of which 20 µL per well was transferred to the assay plate. For the THP1-DUAL assay approximately 100 000 cells per well are seeded in 180 µL medium. The concentration of IFN-γ tested was between 400-6.25 U/mL (2.4-0.04 nM). The assay was incubated at 37°C, 5% CO_2 for 20-24 hours. For the read-out illuminance was measured by the Bio-Rad iMARK microplate reader emission at 465-493 nm. 20 µL of every well was added to a black 96-well plate. The measurement was performed with the following settings: 50 µL of injection volume of Quanti-Luc (#repqlc4lg1, InvivoGen) solution, setting time of 0.1 seconds, measurement interval of 4.00 seconds.

Construct formation

NheI restricted (1x Tango Buffer, 10U NheI) pcDNA3.1$^+$/C-(K)DYK_IL1β (Clone ID: OMu23150D, accession version: NM_008361.4) was used as template for IL-1β amplification by PCR (GC Green Buffer, 0.2 mM dNTPs, 0.1 µM of each primer, 50 ng restricted vector, 2U Phusion polymerase) using primers of **Table 1**. IL-1β fragments were restricted in a two-step protocol with Esp3I/XhoI (IL-1β) or Esp3I/EcoRI (IL-1β-LPETGG), respectively and ligated into NcoI/XhoI (IL-1β) or NcoI/EcoRI (IL-1β-LPETGG) restricted pET28a-vector behind the T7 promoter using T4 DNA Ligase. Ligation products were transformed into *E.coli* XL10 via heat-shock (42°C, 45 seconds) and SOC-medium recovery followed by growth on kanamycin (50 µg/mL) containing LB-agar plates at 37°C.

Table 1 - Primer overview.

Primer Nr.	Construct	Sequence 5'→ 3'
1	IL-1β	TATACCATGGTTCCCATTAGACAACTG
2		AATGCTCGAGTTAGGAAGACACGGA
3	IL-1β-LPETGG-6His	AAAACGTCTCCCATGGTTCCCATTAGACAA
4		CCGGAATTCGGAAGACACGGATTC

Cytokine expression and isolation

Cytokine constructs isolated from *E.coli* XL10 using QIAprep Spin Miniprep Kit were used to transform calcium-competent *E.coli* ArcticExpress (DE3) RP using heat shock (42°C, 45 seconds) and SOC-medium recovery. Colonies were used to inoculate LB-medium containing kanamycin (50 µg/mL) for overnight growth at 37°C, 170 rpm. Overnight culture was diluted 100 times and grown at 37°C, 170 rpm until optical density at 600 nm (OD_{600}) of 0.6 was reached, after which protein expression was induced with addition of 0.5 mM IPTG. Expression took place for 48-72 hours at 10°C, 140 rpm whereafter the bacteria were harvested by centrifugation (30 minutes, 3400*g* at 4°C).

Per 500 mL bacterial culture, 10 mL lysis buffer (50 mM Tris Cl pH 8.0, 25% (w/v) sucrose, 1 mM EDTA, 1 mM PMSF, 2 mM DTT) was applied with 0.4 mg/mL lysozyme. Incubation while gently mixing for 30 minutes at 4°C was followed by adding 10 mM $MgCl_2$, 1 mM $MnCl_2$ and 10U benzonase and 30 minutes incubation at 4°C. The lysate was sonicated (Vibra-cell™ VCX130) for 30 seconds with 5 second pulses with 20% amplitude and 10 second intervals. The lysate was diluted three times with detergent buffer (0.2 M NaCl, 1% (w/v) sodium deoxycholate monohydrate, 1% octylphenoxypolyethoxyethanol (Igepal), 20 mM Tris Cl pH 7.5, 2 mM EDTA, 0.02 mM PMSF, 0.04 mM DTT), incubated for 30 minutes on ice and centrifuged for 20 minutes at 14000*g*, 4°C to collect the supernatant.

Cytokine purification

IL-1β containing lysate was diluted 10 times in 20 mM MES pH 6, incubated overnight at 4°C and centrifuged (10 minutes, 1000*g*, 4°C). The supernatant was loaded (1 mL/min) onto a HiTrap CM sepharose FF column (GE Healthcare Life Sciences) coupled to an Äkta Start System (GE Healthcare Life Sciences) and the protein was eluted using 0-500 mM NaCl in 20 mM MES pH 6. Protein containing fractions were combined, concentrated and buffer exchanged to 20 mM HEPES pH 8 using Amicon® Ultra Filters-15 3K (MERCK #UFC900324). Yields were obtained between 0.5-1.5 mg/mL.

IL-1β-LPETGG containing lysate was first loaded (4 mL/min) onto a HisTrap™ (GE Healthcare Life Sciences) where after the protein was eluted using 0-250 mM imidazole in PBS. IL-1β-LPETGG containing fractions were combined, concentrated and buffer exchanged to 20 mM HEPES pH 8 using Amicon® Ultra Filters-15 3K. Yields were obtained between 0.5-1.5 mg/mL.

Sortase A and VHH-CD11c-SIINFEKL expression and purification

Sortase A and VHH-CD11c-SIINFEKL were expressed and purified as has been described before(**Figure 8A,B**).[59] Briefly, the vector pET28aSrtAΔ59 (kindly provided by Martijn Verdoes of Radboud UMC, #51138 AddGene) was transformed into *E.coli* Rosetta Gami 2 (DE3) via heat shock (42°C, 45 seconds) and SOC-medium recovery. Colonies were grown on kanamycin (50 µg/mL) containing LB-agar plates at 37°C. Colonies were used to inoculate LB-medium containing kanamycin (50 µg/mL) for overnight growth at 37°C, 170 rpm, which was then diluted 50 times in LB-medium and grown at 30°C, 140 rpm until OD_{600}~0.5-0.6. Expression was induced with 1 mM IPTG and took place at 25°C, 140 rpm for 20 hours whereafter the culture was centrifuged for 25 minutes at 3400*g*, 4°C. The resulting pellet was dissolved in 1/10 culture volume nickel binding buffer (50 mM Tris pH 7.5, 150 mM NaCl at room temperature) and centrifuged again. The resulting pellet was stored at -20°C overnight.

Per 500 mL bacterial culture 10 mL ice cold lysis buffer (50 mM Tris pH 7.4 (4°C), 150 mM NaCl, 5 mM $MgCl_2$, 10 mM imidazole, 10% (v/v) glycerol, 1 mg/mL lysozyme, 10U benzonase) was used to dissolve the cell pellet after which the suspension was sonicated on ice for 90 seconds with 30 seconds on/off pulses of 30% amplitude (vibra-cell™ VCX130 of Sonics). The culture was centrifuged (20 minutes, 10 000*g* at 4°C) and the supernatant was passed through a 0.2 µm filter and diluted once in wash buffer (50 mM Tris pH 7.5 (4°C), 150 mM NaCl, 5 mM $MgCl_2$, 10 mM imidazole, 10% (v/v) glycerol) which was then loaded onto 50% HisPur™ Ni-NTA Resin (ThermoFischer Scientific #88222) and incubated for 1 hour, gently mixing at 4°C. Proteins were eluted using 50-500 mM imidazole in storage buffer (50 mM Tris pH 7.5 (4°C), 150 mM NaCl, 10% (v/v) glycerol)(**Figure 8C,D**). Protein containing fractions were combined and buffer exchanged to storage buffer using Amicon® Ultra-15 10K (MERCK UFC901024) filters. Proteins were stored at -80°C.

The pET22b-vector encoding VHH-CD11c-SIINFEKL was transformed into *E.coli* BL21 (DE3) pLysS similar as for sortase A described above. Instead of containing kanamycin, ampicillin (250 µg/mL) and chloramphenicol (25 µg/mL) were used to select for transformed bacteria. VHH-CD11c-SIINFEKL expression and purification were performed as for sortase A.

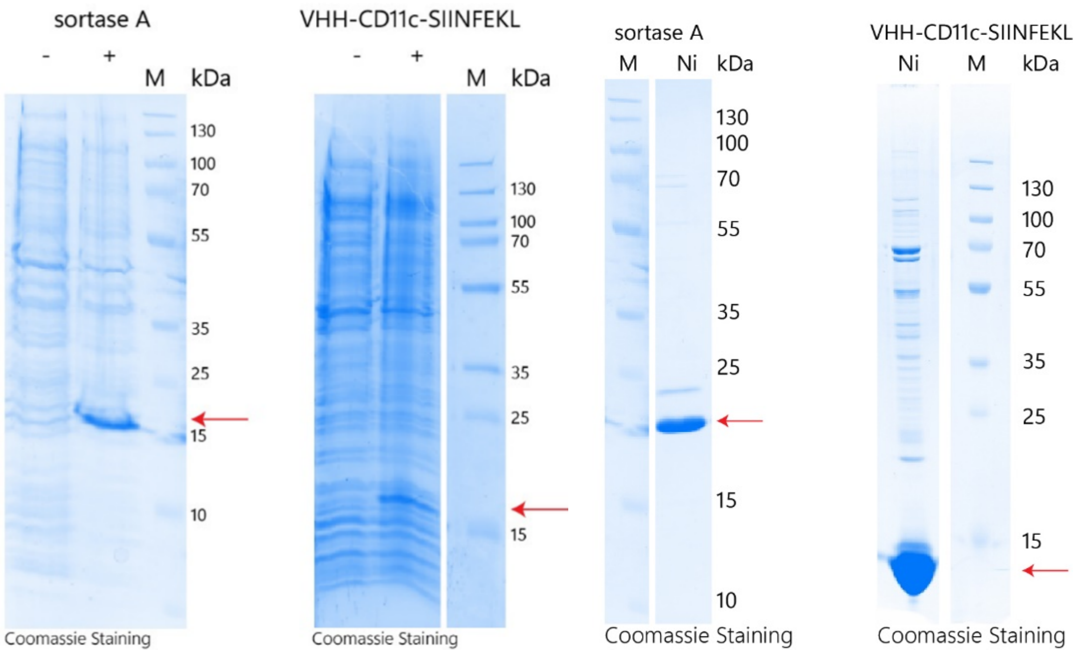

Figure 8 Bacterial expression and purification of sortase A and VHH-CD11c-SIINFEKL using IPTG. *SDS-PAGE analysis of IPTG induced expression of A) Sortase A (~19 kDa) and B) anti-CD11c or VHH-CD11c-SIINFEKL (~15 kDa), by E.coli Rosetta Gami 2 (DE3) and E.coli BL21 (DE3) pLysS, respectively. C) Sortase A (~19 kDa) and D) anti-CD11c or VHH-CD11c-SIINFEKL (~15 kDa) were purified by nickel (Ni) columns. The respective proteins are highlighted with the red arrows.*

Sortase A reaction

VHH-CD11c-SIINFEKL coupling

VHH-CD11c-SIINFEKL was produced with a *N*-terminal thrombin cleavage site protecting the poly-glycine *N*-terminus. Using the 25x diluted thrombin (Merck #69671-3) with 10 µg protein in 50 µL total volume released the *N*-terminal poly-glycine tail. Caging took place prior to the reaction with sortase and nanobody. Sortase reaction was performed with 4 µM IL-1β-LPETGG-6His, 3 µM sortase A, 8-10 µM VHH-CD11c-SIINFEKL in sortase buffer (50 mM Tris pH 7.5, 150 mM NaCl, 10% (v/v) glycerol, 4°C) supplemented with 1 mM $CaCl_2$. Incubation took place for 15 minutes at 37°C, 300 rpm.

Purification IL-1β-VHH-FR-SIINFEKL

Coupled protein was concentrated to a final volume of maximal 500 µL before loading onto a Superdex™ 75 increase 10/300 GL column connected to Äkta start system, equilibrated with 20 mM HEPES pH 8. Fractions of 500 µL IL-1β-VHH-CD11c-SIINFEKL were separated from the starting materials. Fraction analysis was performed using SDS-PAGE with coomassie staining. Pure fractions were combined and stored at -80°C.

Statistical analysis

All data were normalised using Microsoft Excel. All data are reported as mean ± SEM of n=3-7 independent experiments. Statistical analysis was carried out using R 4.1.0 (R Core Team, 2021), the base, graphics, methods, stats, utils, and ggplot2 packages. Parametric tests, one-way ANOVA, or Brown-Forsythe ANOVA tests were used for data with normal distribution, based on the homoscedasticity of the data, verified with Bartlett's test. If the omnibus test had a $p<0.05$, unpaired t-test with or without Welch's correction (Welch's correction applied to different SD pairs) post hoc tests were performed based on the F-test. For data not normally distributed, non- parametric Kruskal–Wallis comparisons were carried out with Mann-Whitney post hoc comparisons test. Results were considered significant when $p<0.05$. Graphs were made using GraphPad Prism 8.4.3.
